# AI-driven diagnosis of mpox using deep learning models

**DOI:** 10.1371/journal.pone.0352161

**Published:** 2026-07-10

**Authors:** Bassam W. Aboshosha, Shafiq Ul Rehman, Lamees N. Mahmoud, Ibrahim Sadek

**Affiliations:** 1 School of Engineering and Computer Science, University of Hertfordshire, Hosted by Global Academic Foundation, New Administrative Capital, Egypt; 2 College of Information Technology, Kingdom University, Riffa, Kingdom of Bahrain; 3 Biomedical Engineering Department, Faculty of Engineering, Helwan University (formerly Helwan University), Helwan, Egypt; Federal University Otuoke, NIGERIA

## Abstract

Mpox lesions can resemble other dermatological conditions, motivating image-based screening, yet published studies remain difficult to compare owing to differences in dataset construction, augmentation policy, and evaluation design. This study provides a leakage-aware benchmark for binary mpox classification using a unified dataset assembled from MSLD v1.0 and v2.0. Seven pretrained backbones and a weighted ensemble were compared under group-stratified five-fold cross-validation with original-only test evaluation, validation-based threshold selection, and temperature scaling. The weighted ensemble achieved mean accuracy 0.8729, F1-score 0.8334, and AUC 0.9388; ConvNeXt-Tiny was the strongest single model (F1 0.8159, AUC 0.9284). These grouped original-only results are intentionally conservative relative to augmentation-heavy or single-split designs and should be interpreted as deflated but more trustworthy reference values. Post hoc calibration analysis, content-level near-duplicate auditing, and a test-time augmentation ablation are provided to substantiate the methodological claims. The contribution is methodological: a transparent benchmark emphasizing reproducible dataset curation, grouped evaluation, and calibrated comparison, while highlighting the limitations of current public skin-image data. Accordingly, these results should be interpreted as a reproducible reference benchmark rather than a clinically validated diagnostic tool, and external clinical validation remains necessary before deployment.

## 1 Introduction

Mpox is a zoonotic disease caused by monkeypox virus (MPXV), an orthopoxvirus related to variola virus and other members of the same genus. Typical manifestations include fever, headache, myalgia, lymphadenopathy, and vesiculopustular skin lesions, but diagnosis based on clinical appearance alone may be difficult because mpox lesions can resemble other rash-associated conditions such as chickenpox, measles, and other vesicular or pustular disorders [1,2]. Laboratory confirmation therefore remains central to case verification, and lesion-derived material analysed by nucleic acid amplification testing, particularly PCR-based testing, is regarded as the reference diagnostic approach [2]. At the same time, the visual nature of many mpox manifestations has motivated interest in image-based decision-support systems as adjunct tools for preliminary screening in settings where rapid specialist assessment may be limited.

Artificial intelligence, and especially deep learning, has been increasingly explored for mpox-related tasks, ranging from public-health surveillance through social-media sentiment analysis [3] to diagnostic imaging from skin lesions. Chadaga et al. systematically reviewed artificial intelligence applications for mpox and identified diagnostic imaging as one of the main research directions in the field [4]. Early lesion-image studies showed that pretrained models could provide useful discrimination on public datasets [5,6], while later studies expanded the comparison space through transfer learning on multiple datasets and ensemble learning [7,8]. These studies established the feasibility of computer-aided mpox screening from lesion images, but they also exposed a persistent problem: model results are difficult to compare directly because datasets, augmentation policies, class definitions, and validation protocols vary substantially across papers.

Recent work has made this limitation explicit. Hossain et al. argued that poor dataset quality, weak generalizability, lesion variability, image noise, unsuitable augmentation, and inconsistent benchmarking remain major barriers to reliable mpox diagnosis from skin images [9]. This concern is reinforced by Vega et al. who showed that some early publicly shared mpox skin-image datasets contained flawed or medically irrelevant web-scraped content, underscoring the need for explicit curation and evaluation rules [10]. For this reason, the key need is not simply another high headline accuracy, but a benchmark in which dataset assembly, group handling, training policy, threshold selection, and evaluation are documented clearly enough to permit defensible interpretation.

The methodological choice of transfer learning with pretrained backbones is motivated by three practical considerations specific to the mpox imaging domain. First, the total number of publicly available original mpox lesion images remains small (on the order of a few hundred), which makes training deep architectures from scratch prone to severe overfitting; transfer learning from ImageNet-pretrained weights mitigates this by providing a robust feature initialization that generalizes well to medical texture and colour cues with limited fine-tuning data [4]. Second, different backbone families encode complementary inductive biases: convolutional networks such as ConvNeXt-Tiny and MobileNetV3-Large emphasize local texture and spatial hierarchy, whereas vision transformers such as ViT-B/16 capture long-range dependencies across the image. The extent to which these biases are advantageous for lesion recognition under heterogeneous acquisition conditions is an empirical question that the present benchmark is designed to address. Third, weighted ensembling is included because prior mpox studies have shown that combining diverse classifiers can improve robustness when no single model dominates across all operating metrics [5,8], and the present protocol provides a controlled setting to quantify this gain under stricter evaluation conditions.

Against this background, the present study investigates binary mpox image classification using a unified curated dataset assembled from MSLD v1.0 and MSLD v2.0 under explicit selection and class-mapping rules. The benchmark compares seven pretrained backbones and a weighted ensemble under a common protocol that emphasizes group-aware splitting, evaluation on original images only, controlled use of augmented data during training, validation-based threshold selection, and fold-wise reporting.

The objectives of this study are threefold. First, it aims to construct a unified curated binary dataset for mpox skin-image classification with fully specified class-mapping and selection rules. Second, it aims to compare multiple pretrained transfer-learning models and an ensemble strategy under a shared training and evaluation pipeline. Third, it aims to examine how dataset construction and leakage-aware evaluation influence the interpretation of performance on public mpox lesion data. The contribution is therefore methodological rather than architectural: the study does not introduce a new backbone, but instead provides a unified curation recipe, a leakage-aware validation design, and fold-wise reference results intended to support more defensible comparison with prior and future mpox skin-image studies.

Accordingly, the study addresses the following research questions:

- **RQ1.** Under a shared group-aware evaluation protocol, which pretrained backbone provides the most reliable performance for binary mpox image classification?
- **RQ2.** Does weighted ensembling provide a consistent advantage over individual backbones when testing is restricted to original images and summarized across cross-validation folds?
- **RQ3.** What do the results reveal about the current strengths and limitations of publicly available mpox skin-image datasets for reproducible artificial-intelligence benchmarking?

## 2 Related work

Recent peer-reviewed work on mpox image analysis has focused mainly on transfer learning with pretrained convolutional neural networks, with some studies extending this direction toward mobile deployment, customized hybrid models, dataset curation, and ensembling [4]. Because the literature spans binary classification, multiclass differential diagnosis, and even broader mpox-related artificial-intelligence applications, direct comparison of headline accuracies requires careful attention to task definition, dataset composition, augmentation policy, and validation design.

Among the early lesion-image studies, Sitaula and Shahi compared 13 pretrained deep-learning models and then combined selected models using majority voting; their ensemble achieved 85.44% precision, 85.47% recall, 85.40% F1-score, and 87.13% accuracy on a publicly available lesion dataset [5]. Sahin et al. developed a mobile screening application and reported 91.11% test accuracy, together with average inference times of 197 ms, 91 ms, and 138 ms on three mobile devices [6]. These studies established the feasibility of mpox screening from skin images, but they were based on early public datasets and relatively simple binary pipelines.

Subsequent studies diversified both the datasets and the modelling strategies. MonkeyNet, introduced by Bala et al., combined a newly compiled multiclass lesion dataset with a modified DenseNet-201 and reported 93.19% accuracy on the original data and 98.91% on the augmented data [11]. Altun et al. reported that a customized MobileNetV3-s transfer-learning model achieved an average F1-score of 0.98, AUC of 0.99, and accuracy of 0.96 [12]. Uysal proposed a hybrid deep-learning system that combined high-performing CNNs with LSTM and reported 87% test accuracy with Cohen’s kappa of 0.8222 on a four-class dataset [13]. Azar et al. evaluated seven deep neural networks under both two-class and four-class settings; their DenseNet201-based architecture reached 97.63% accuracy in the two-class scenario and 95.18% in the four-class scenario [14].

Other studies emphasized explainability, efficient deployment, or broader dataset coverage. Thieme et al. developed a deep-learning algorithm for classifying mpox skin lesions and validated it on a geographically diverse clinical dataset, achieving strong discrimination and highlighting the potential for AI-assisted triage in clinical settings [15]. Nayak et al. reported an average accuracy of 91.19% and an Mpox-class F1-score of 92.55% in a four-class explainable-AI study based on residual networks and SqueezeNet [16]. In a related study using five efficient CNN backbones, the same group reported 99.49% validation accuracy for a ResNet-18-based classifier on a public binary dataset and highlighted the suitability of the models for smartphone-like devices [17]. Almufareh et al. evaluated transfer learning on two public lesion datasets and showed that model ranking changed across datasets: MobileNetV2 achieved the best accuracy on MSID (0.96), whereas InceptionV3 achieved the best accuracy on MSLD (0.9333) [7]. Pramanik et al. proposed an amalgamation of CNN models aided with Beta function-based normalization for mpox detection from skin lesion images and reported strong classification performance on a public dataset [18]. Muñoz-Saavedra et al. extended the field toward ensemble learning and reported that single networks reached 93% accuracy whereas a three-network ensemble reached 98.33% accuracy in a three-class setting [8]. Bamaqa et al. later combined transfer learning with Sparrow Search Algorithm optimization and reported 99.87% accuracy for an optimized VGG19 model on two public datasets, although their binary task merged multiple pox-like conditions into a single positive class [19]. Aslam et al. proposed a deep transfer-learning approach for mpox recognition from visual images, further demonstrating the viability of pretrained networks in this domain [20].

Table 1 summarizes representative peer-reviewed studies together with the task setting, dataset context, validation strategy, best reported result, and the main reason why direct comparison with the present benchmark remains limited. Reporting these elements together is essential because published headline figures often depend strongly on class structure, augmentation policy, and whether performance is reported on validation or held-out test data.

**Table 1:** Standardized comparison of representative peer-reviewed mpox skin-image studies.

Study	Task / dataset	Split / validation	Best reported result	Comparability limitation
Sitaula and Shahi (2022) [5]	Binary mpox/non-mpox classification using 13 pretrained models plus an ensemble. Dataset: 1 754 augmented images from four categories (587 mpox, 552 normal, 329 chickenpox, 286 measles), later remapped to binary.	Random 5-fold cross-validation.	Ensemble: precision 85.44%, recall 85.47%, F1 85.40%, accuracy 87.13%.	Binary evaluation performed on an augmented four-class pool remapped to two classes; data-generation policy differs from the present benchmark.
Sahin et al. (2022) [6]	Binary mobile screening on MSLD. Original set: 228 images (102 mpox, 126 non-mpox), expanded by augmentation to 1 428 mpox and 1 764 non-mpox images.	Single predefined Fold1 split provided by the MSLD repository; original images divided at approximately 70/10/20 for training, validation, and test with patient independence maintained.	Test accuracy 91.11%; mobile inference times 197, 91, and 138 ms.	Mobile deployment study evaluated on a single early split rather than repeated cross-validation.
Bala et al. (2023) [11]	Four-class classification (chickenpox, measles, mpox, normal). Original dataset: 770 images; augmented dataset: 8 689 images.	80:20 training-to-test split; 20% of the training portion reserved for validation.	Original data: 93.19%. Augmented data: 98.91%.	Multiclass design and separate original/augmented reporting make the headline accuracy non-comparable to a binary original-only benchmark.
Altun et al. (2023) [12]	Binary optimized transfer-learning CNN on a custom web-scraped dataset. Total: 2 056 images; training: 1 742; validation: 158; test: 156.	Held-out test set; a validation subset was drawn from training data during hyperparameter optimization.	Optimized hybrid MobileNetV3-s: average F1 0.98, AUC 0.99, accuracy 0.96, recall 0.97.	Custom hybrid architecture and custom web-scraped dataset; no shared public benchmark protocol.
Uysal (2023) [13]	Four-class hybrid CNN-LSTM study. Original dataset: 770 images (293 normal, 279 mpox, 91 measles, 107 chickenpox); augmented and balanced to 1 200 images.	80/10/10 training, validation, and test after preprocessing and augmentation.	Test accuracy 87%; Cohen’s kappa 0.8222.	Balanced multiclass augmented dataset and hybrid CNN-LSTM architecture differ markedly from the current binary grouped protocol.
Azar et al. (2023) [14]	Two-class and four-class lesion classification using seven DNNs. Dataset: 1 710 images from four classes; both binary remapping and four-class analysis were reported.	10-fold cross-validation with hyperparameter optimization.	Two-class: DenseNet201 97.63%. Four-class: 95.18%.	Different source dataset; two task formulations evaluated within a single study complicate single-point comparison.
Nayak et al. [16]	Four-class explainable-AI study using ResNet-50, ResNet-18, ResNet-10, and SqueezeNet on MSID. Dataset: 770 images (107 chickenpox, 91 measles, 279 mpox, 293 normal).	Training and validation splits used; the paper reports peak validation accuracy of 95.42% for the best trial. The exact fold count or hold-out ratio is not clearly recoverable from the accessible full text.	Average accuracy 91.19%; mpox-class F1 92.55%.	Class-wise F1 in a four-class explainable-AI setting is not directly comparable with global binary metrics.
Nayak et al. [17]	Binary classification using five pretrained networks on MSLD. Dataset: 228 images (102 mpox, 126 others) resized to 224×224.	Training and validation split with hyperparameter tuning; reported 99.49% is a *validation* accuracy (all models reached 100% training accuracy). No separate held-out test set is described in the accessible full text.	ResNet-18 validation accuracy 99.49%; sensitivity 99.43%, specificity 100%.	Headline accuracy is a validation figure obtained during hyperparameter search; absence of a leakage-aware held-out test split limits comparability.
Almufareh et al. (2023) [7]	Binary transfer learning on two public datasets. MSLD: 228 images (102 mpox, 126 others). MSID: 477 images after binary remapping (279 mpox, 198 others); note that the original MSID contains 770 four-class images, and the 477-image count reflects the subset reported by the authors after their specific preprocessing.	Augmentation applied; early stopping used during training. The specific training, validation, and test split ratio is not reported in the accessible full text.	MSID, MobileNetV2: accuracy 0.96, balanced accuracy 0.9655. MSLD, InceptionV3: accuracy 0.9333, balanced accuracy 0.94.	Model ranking reverses across datasets, limiting any single leaderboard-style comparison; split protocol not recoverable.
Muñoz-Saavedra et al. (2023) [8]	Three-class classification (mpox, healthy, other skin disease). Custom public dataset: 300 images total, balanced at 100 images per class.	60:20:20 training, validation, and test split.	Single network: 93%. Three-network ensemble: 98.33%.	Three-class custom dataset with its own collection and balance rules; not a direct binary comparator.
Bamaqa et al. (2024) [19]	Binary normal-vs.-pox classification on two public datasets (42 162 images total). The positive class pooled mpox, chickenpox, smallpox, cowpox, and measles into one “pox” label.	Comparative optimization study over multiple search strategies. A patient-aware or grouped split protocol is not stated in the indexed abstract or highlights.	SpaSA-optimized VGG19: accuracy 99.87%.	Positive class is broader than mpox alone; optimization target differs from a standard mpox-only lesion benchmark.
Elhadidy et al. (2025) [21]	Multiclass benchmarking on MSLD v2.0 using five pretrained CNN and Transformer backbones. The study used the single-source six-class MSLD v2.0 dataset with augmentation to improve robustness.	The accessible abstract reports validation accuracy after augmentation-based training, but the exact split design is not fully recoverable from the indexed source text.	Xception: 99.92% validation accuracy.	Single-source multiclass benchmark centered on validation accuracy; it does not address cross-source unification or original-only grouped evaluation.
**Present study (2025)**	Binary classification on a unified dataset from MSLD v1.0 and v2.0. 876 original images (339 mpox, 537 non-mpox); 1 357 with augmented. Seven pretrained backbones + weighted ensemble.	Group-stratified 5-fold cross-validation on original images only. Augmented images used during training only. Validation-based threshold selection and temperature scaling per fold.	Weighted ensemble: F1 0.8334 ± 0.0432, AUC 0.9388 ± 0.0203. Best single model (ConvNeXt-Tiny): F1 0.8159 ± 0.0727.	Intentionally conservative protocol; results are lower than augmentation-heavy single-split studies but are designed for reproducibility and cross-study comparability.

Taken together, the literature shows that mpox image classification is technically feasible and that strong performance can be obtained under several settings. At the same time, public datasets remain heterogeneous, and recent critique has made clear that benchmarking practices are not yet sufficiently standardized [9,10]. Recent MSLD v2.0 benchmarking work also continues to report very high validation accuracy under single-source, augmentation-based protocols [21]. The present study is positioned within this gap. Rather than proposing a new architecture, it focuses on transparent dataset construction from MSLD v1.0 and MSLD v2.0, explicit group handling, original-only test evaluation, within-fold calibration, and fold-wise reporting under a shared protocol, thereby providing a clearer basis for comparing transfer-learning backbones and weighted ensembling on a unified curated binary benchmark.

## 3 Materials and methods

### 3.1 Study design

This study was designed as a reproducible benchmarking and methodological-validation study for binary mpox skin-image classification using transfer learning. The benchmark combined multiple public lesion-image resources under explicit class-mapping rules and evaluated all models using group-stratified 5-fold cross-validation. Augmented images were allowed during training under controlled sampling, but all validation-driven model selection and all reported test metrics were tied to original-image evaluation only. Post hoc temperature scaling was applied within each fold, and a weighted ensemble was optimized on validation predictions. The study should therefore be interpreted as a validation-oriented benchmarking exercise: its primary aim is to reduce optimistic bias and improve comparability across models, not to claim a novel diagnostic architecture.

### 3.2 Unified dataset construction

The unified benchmark incorporated MSLD v1.0 and MSLD v2.0 [7,22]. For MSLD v1.0, the binary mapping followed the folder structure Monkey Pox versus Others for original images and Monkeypox_augmented versus Others_augmented for augmented images. For MSLD v2.0, only the Monkeypox class was mapped to the positive label, whereas Chickenpox, Cowpox, Healthy, HFMD, and Measles were merged into the negative label. To avoid contaminating the benchmark with a predefined external test split, only the Train and Valid subsets of MSLD v2.0 were used in the reported experiments.

Source prefixes (v1__ and v2__) were attached during dataset materialization to preserve provenance and avoid filename collisions. The final original-image pool used to define cross-validation folds contained 876 images, corresponding to 339 mpox and 537 non-mpox images. After controlled inclusion of augmented images, the combined pre-fold pool of original and augmented images contained 1,357 samples, of which 876 were original. Table 2 summarizes the unified dataset construction, class mapping, and inclusion rules.

**Table 2:** Unified dataset construction rules.

Source	Positive mapping	Negative mapping	Inclusion rule
MSLD v1.0 [7]	Monkey Pox / Monkeypox augmented	Others / Others augmented	All readable files in the designated folders
MSLD v2.0 [22]	Monkeypox	Chickenpox, Cowpox, Healthy, HFMD, and Measles	Only the Train and Valid subsets from folds 1–5

### 3.3 Leakage control, grouping, and split policy

Data splitting was based on original images only. Augmented images were excluded from fold construction and from all reported test evaluations. This design ensured that the benchmark never reported final performance on synthetic variants. During dataset preparation, augmented images were capped at three variants per inferred group (max_aug_per_group = 3), following the common practice of limiting augmentation multiplicity to avoid overfitting to synthetic variants [23], and the training mixture was downsampled so that original images represented approximately 70% of the combined training pool (train_orig_ratio = 0.7), a ratio chosen to preserve the distributional characteristics of the original acquisition conditions while still benefiting from augmented diversity. In addition to filename-based grouping, a post hoc content-level near-duplicate audit was conducted using 64-bit perceptual hashing (pHash) computed over all 876 original images. Image pairs whose Hamming distance fell at or below a conservative threshold of 10 (out of 64 bits, corresponding to approximately 84% bit agreement) were flagged as candidate near-duplicates; this threshold is consistent with the range of 8–12 commonly used in perceptual-hash-based duplicate detection [24]. The audit identified 95 candidate pairs; the vast majority (more than 80) had a Hamming distance of exactly zero, indicating perceptually identical content. Inspection revealed that nearly all pairs consist of the same image present in both MSLD v1.0 and v2.0 under different filename conventions (e.g., v1__M01_04.jpg ↔ v2__MKP_01_04.jpg), confirming that the two dataset versions share a substantial overlapping core. Three additional intra-source near-duplicate pairs were detected within MSLD v2.0 itself (CWP_02 ↔ CWP_31, HEALTHY_21 ↔ HEALTHY_77, HFMD_130 ↔ HFMD_96). Crucially, in every one of the 95 identified pairs both members belonged to the same filename-derived group and therefore to the same cross-validation fold, yielding zero cross-fold leaks. Leakage control therefore rests on three complementary layers: source prefixing, filename-based grouping with original-only fold construction, and verified content-level deduplication.

The reported experiments used group_stratified splitting with 5 folds and an internal validation fraction of 0.20. Group identifiers were inferred with a filename-based regular expression that stripped optional source prefixes (for example, v1__) together with augmentation suffixes such as _ORIGINAL and numeric variant tags. This grouping rule kept related filenames within the same fold. The final run identified 617 groups, with an average of 1.42 images per group and a maximum of 14 images per group. A filename-based leakage sanity check indicated that no derived leakage identifier was present in more than one split during the reported cross-validation run.

### 3.4 Compared models

Seven pretrained backbones were evaluated: EfficientNetV2-S [25], ConvNeXt-Tiny [26], ViT-B/16 [27], ResNet-50 [28], DenseNet-121 [29], EfficientNet-B0 [30], and MobileNetV3-Large [31]. All models were trained at an input resolution of 224×224 pixels.

### 3.5 Shared training and evaluation protocol

All experiments used a batch size of 16, a maximum of 30 epochs, mixed-precision training, two data-loading workers, and a weighted sampler. The shared training mode was original+aug, whereas validation and test evaluation used original images only. Online image augmentation was disabled because the benchmark already included a curated augmented-image pool. After the main training stage, each model was fine-tuned on original images only for 5 epochs at a learning rate of $1\times 10^{-5}$.

Early stopping used f1_at_precision as the monitored metric, with patience set to 4 epochs. Validation-based threshold selection used the same f1_at_precision strategy with a precision target of 0.80 and a minimum recall target of 0.85. Probability calibration was performed by temperature scaling [32] fitted on the validation subset of each fold. At test time, 16-fold test-time augmentation was enabled for all backbones. Table 3 provides a compact summary of the shared training and evaluation settings.

**Table 3:** Shared training and evaluation settings.

Setting	Value
Cross-validation folds	5
Validation fraction within each fold	0.20
Split strategy	Group-stratified
Splitting base	Original images only
Training mix	Original + augmented
Original-image ratio in training mix	0.70
Maximum augmented variants per group	3
Input size	224×224
Batch size	16
Maximum epochs	30
Fine-tuning stage	Original-only
Fine-tuning epochs / learning rate	5 / $1\times 10^{-5}$
Mixed precision	Enabled
Weighted sampler	Enabled
Threshold strategy	f1_at_precision
Precision target / minimum recall	0.80 / 0.85
Calibration	Temperature scaling
Test-time augmentation	16-fold

### 3.6 Multi-objective hyperparameter selection

Backbone-specific hyperparameters were selected using multi-objective optimization (MOO) based on Optuna/NSGA-II [33]. The three objectives were to maximize AUC, maximize F1 at the precision-constrained threshold, and minimize log loss. The search used a population size of 8, 3 generations, and 6 evaluation epochs, corresponding to 24 trials per backbone and per fold. Feasibility constraints required precision ≥ 0.80, AUC ≥ 0.80, and F1 ≥ 0.80, and candidate selection used the f1_at_precision_first rule.

The tuned search space included learning rate, weight decay, label smoothing, MixUp alpha, CutMix alpha, loss type, focal-loss alpha and gamma, optimizer, scheduler, warmup duration, drop-path rate, gradient clipping norm, and classifier-head learning rate. To avoid notation ambiguity, the subscripted symbols $\alpha_{f}$ and $\gamma_{f}$ in Table 4 denote the focal-loss class-weight and focusing parameters, respectively, whereas $\alpha_{m}$ and $\alpha_{c}$ in Table 5 denote the Beta-distribution concentration parameters for MixUp and CutMix augmentation. In the reported run, the same configuration was selected for a given backbone in all five folds, so Tables 4 and 5 report the exact selected settings used during cross-validation. The exported tuning records assigned identical selected settings to ResNet-50 and DenseNet-121 in this run; these values are reported verbatim from the recorded outputs obtained under the shared search space and selection rule.

**Table 4:** Selected backbone-specific optimization and loss hyperparameters.

Model	LR	WD	Label smooth.	Loss	Focal $\alpha_{𝐟}$	Focal $\gamma_{𝐟}$	Optimizer	Scheduler
EfficientNetV2-S	3.9 038 × 10^-4^	1.2 503 × 10^-5^	0.0701	focal	0.3221	2.4771	AdamW	plateau
ConvNeXt-Tiny	3.0 550 × 10^-5^	3.8 194 × 10^-6^	0.0701	focal	0.3531	2.1184	AdamW	plateau
ViT-B/16	3.0 550 × 10^-5^	1.0 569 × 10^-5^	0.0940	focal	0.4070	2.1184	Adam	plateau
ResNet-50	5.9 540 × 10^-5^	1.3 315 × 10^-4^	0.0547	ce	0.3256	1.6350	Adam	none
DenseNet-121	5.9 540 × 10^-5^	1.3 315 × 10^-4^	0.0547	ce	0.3256	1.6350	Adam	none
EfficientNet-B0	1.0 645 × 10^-3^	5.4 870 × 10^-5^	0.1215	focal	0.2878	2.2738	Adam	warmup_cosine
MobileNetV3-Large	3.9 038 × 10^-4^	3.1 237 × 10^-4^	0.0701	focal	0.3531	2.4771	AdamW	plateau

**Table 5:** Selected backbone-specific augmentation and regularization hyperparameters.

Model	MixUp $\alpha_{𝐦}$	CutMix $\alpha_{𝐜}$	Warmup ep.	Drop path	Grad clip	Head LR
EfficientNetV2-S	0.1302	0.1759	1.0079	0.1053	1.0	6.3 680 × 10^-4^
ConvNeXt-Tiny	0.1302	0.1759	0.9835	0.1919	1.0	6.3 680 × 10^-4^
ViT-B/16	0.0345	0.0572	0.0410	0.1919	1.0	1.6 317 × 10^-4^
ResNet-50	0.0624	0.0709	0.3972	0.1863	0.5	2.7 253 × 10^-4^
DenseNet-121	0.0624	0.0709	0.3972	0.1863	0.5	2.7 253 × 10^-4^
EfficientNet-B0	0.0384	0.0876	1.2190	0.0300	1.0	1.8 068 × 10^-3^
MobileNetV3-Large	0.1302	0.1759	0.9835	0.1053	1.0	6.3 680 × 10^-4^

### 3.7 Weighted ensembling

In addition to single-model evaluation, the benchmark included a weighted probability ensemble. Ensemble weights were optimized by random search using 4,096 trials and the same precision-constrained F1 criterion used for threshold selection. The ensemble was evaluated only after all component backbones had completed their fold-specific model selection, fine-tuning, and calibration steps.

### 3.8 Evaluation metrics

Performance was summarized across the five folds using six complementary metrics. For a binary confusion matrix with true positives (TP), false positives (FP), true negatives (TN), and false negatives (FN):


$$\begin{array}{cc} \text{Accuracy} & =\frac{\text{TP}+\text{TN}}{\text{TP}+\text{FP}+\text{TN}+\text{FN}},\\ \text{Precision} & =\frac{\text{TP}}{\text{TP}+\text{FP}},\\ \text{Recall} & =\frac{\text{TP}}{\text{TP}+\text{FN}},\\ \text{Specificity} & =\frac{\text{TN}}{\text{TN}+\text{FP}},\\ \text{F1-score} & =\frac{2\times \text{Precision}\times \text{Recall}}{\text{Precision}+\text{Recall}}. \end{array}$$

The area under the receiver operating characteristic curve (AUC) was computed from the predicted probability outputs using the standard trapezoidal-rule implementation in scikit-learn (roc_auc_score) [34] and is not restated as a closed-form expression above. Each metric was computed independently within each of the five test folds, and the reported values represent the mean ± standard deviation across folds. To better characterize the operating behaviour, the raw confusion-matrix components (TP, FP, TN, FN) were also aggregated as mean ± standard deviation across folds.

### 3.9 Statistical analysis

To determine whether observed performance differences between models reflect genuine architectural effects rather than sampling variability, four complementary pairwise statistical tests were applied within each fold. McNemar’s test [35] assessed the significance of disagreements between two models’ hard-label predictions. A permutation test with 2,000 random permutations evaluated differences in F1-score. The Wilcoxon signed-rank test [36] was applied to per-sample predicted-probability differences. DeLong’s test [37] was used to compare AUC values. All *p*-values were corrected for multiple comparisons using the Bonferroni method across all pairwise model combinations within each fold. Because the statistical test suite included an additional ensemble variant not reported in the main results, the number of compared methods was nine in four folds and eight in one fold, yielding 36 and 28 pairwise comparisons respectively (adjusted α=0.05/36≈0.0014 or 0.05/28≈0.0018). Results are reported in Section 4.2.

### 3.10 Implementation details

All experiments were implemented in Python 3.10 using PyTorch 2.1 and torchvision 0.16 for model definition, pretrained weight loading, and training. Pretrained ImageNet-1K weights were obtained from the torchvision model zoo using the default IMAGENET1K_V1 weight variants for each backbone. Hyperparameter optimization used Optuna 3.4 with the NSGA-II sampler. Temperature scaling, threshold selection, ensemble weight optimization, and Expected Calibration Error computation were performed using scikit-learn 1.3 and SciPy 1.11. Content-level near-duplicate auditing used 64-bit perceptual hashing via the ImageHash library. All training and primary inference were conducted on a single NVIDIA A100 (40 GB) GPU; the TTA ablation re-evaluation (Section 4.7) was conducted on an NVIDIA GeForce RTX 4060 Laptop GPU using the same saved checkpoints. Each backbone completed five-fold training, validation, and test evaluation in approximately 20–45 minutes depending on model size, corresponding to a total compute budget of approximately 15–25 GPU-hours including MOO trials (840 trial-runs at 6 epochs each) and ensemble optimization. The random seeds for each fold were fixed to 12345–12349 to ensure reproducibility; group-stratified fold assignment is deterministic given the file list ordering and seed. The complete run configuration, command log, and fold-wise outputs are supplied as supplementary material and are also available in the project repository.

### 3.11 Ethics statement

This study used publicly available, anonymized image data and did not require institutional ethical approval.

## 4 Results

### 4.1 Overall comparative performance

**Fig 1:**
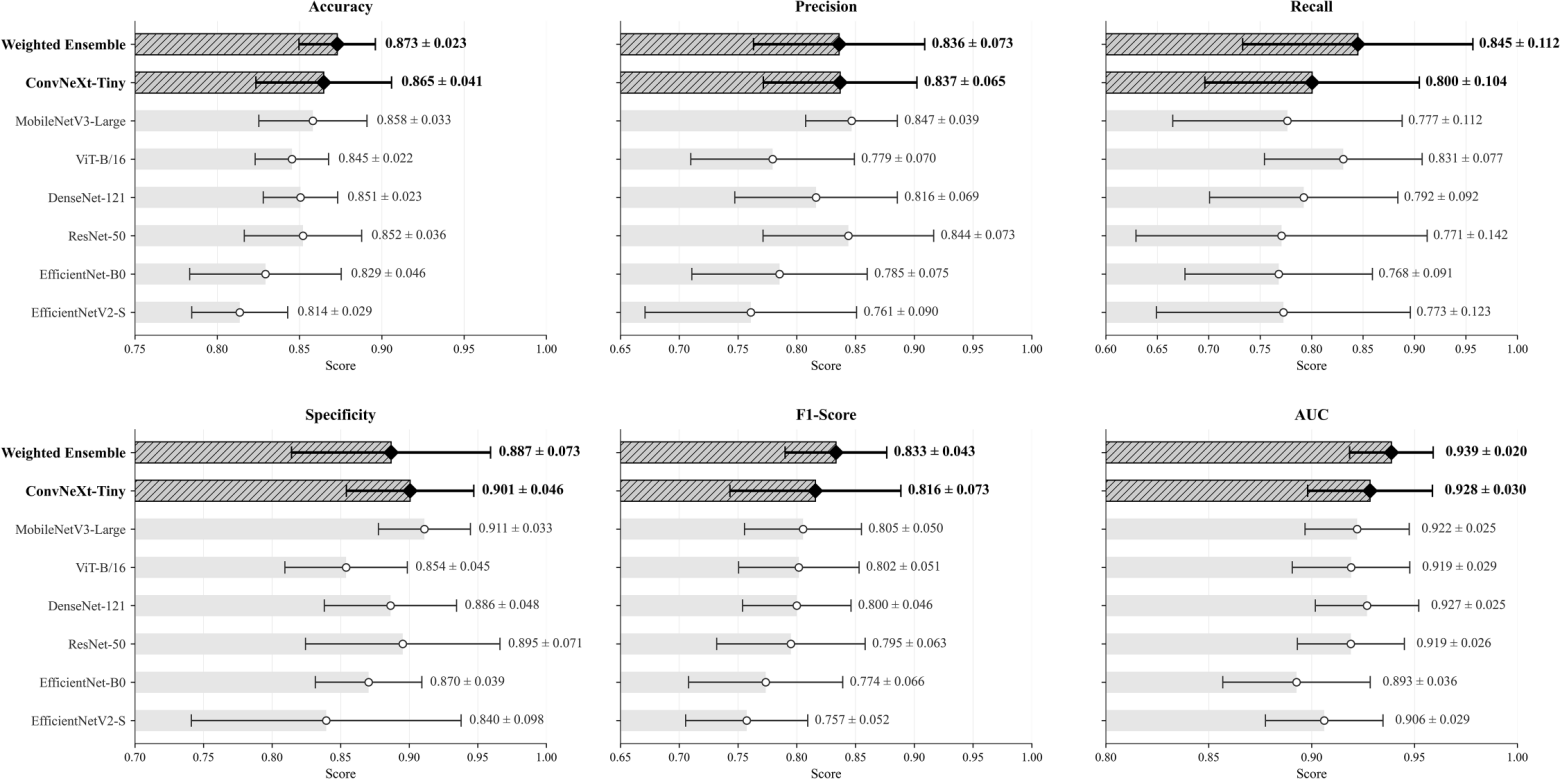
Cross-validation performance summary of the evaluated methods across five folds. Each point represents the mean value across the five cross-validation folds, and the whiskers represent ±1 standard deviation around that mean. Methods are ordered by mean F1-score.

Table 6 and Fig 1 summarize fold-wise test performance for all evaluated models. All reported mean ± standard deviation values reflect inter-fold test-partition variability across the five cross-validation splits (not training stochasticity, since each fold uses a deterministic seed). As a reference, the majority-class baseline (always predicting “Others”) would yield an accuracy of 0.613 given the class distribution (339 mpox, 537 non-mpox), confirming that all evaluated models provide substantial improvement over the trivial baseline. The weighted ensemble achieved the best overall performance, with accuracy of 0.8729 ± 0.0232, precision of 0.8360 ± 0.0728, recall of 0.8449 ± 0.1119, F1-score of 0.8334 ± 0.0432, and AUC of 0.9388 ± 0.0203. For comparison, evaluating the same weighted ensemble at the fixed default threshold of 0.5 (without validation-based threshold selection) yielded a mean F1-score of 0.8255, indicating that the precision-constrained threshold strategy improved F1-score by +0.008 on average. Among the individual backbones, ConvNeXt-Tiny provided the strongest overall balance, achieving 0.8159 ± 0.0727 F1-score and 0.9284 ± 0.0303 AUC. ViT-B/16 yielded the highest mean recall among the individual backbones, whereas MobileNetV3-Large yielded the highest mean precision and specificity among the single models.

**Table 6:** Fold-wise test performance (mean ± standard deviation) across five cross-validation folds.

Model	Acc.	Prec.	Rec.	Spec.	F1	AUC
ConvNeXt-Tiny	0.8647 ± 0.0412	0.8369 ± 0.0654	0.8005 ± 0.1043	0.9007 ± 0.0465	0.8159 ± 0.0727	0.9284 ± 0.0303
DenseNet-121	0.8506 ± 0.0226	0.8164 ± 0.0692	0.7923 ± 0.0915	0.8863 ± 0.0482	0.8000 ± 0.0462	0.9270 ± 0.0251
EfficientNet-B0	0.8293 ± 0.0460	0.7854 ± 0.0746	0.7680 ± 0.0911	0.8704 ± 0.0389	0.7736 ± 0.0656	0.8927 ± 0.0358
EfficientNetV2-S	0.8137 ± 0.0291	0.7610 ± 0.0900	0.7726 ± 0.1235	0.8395 ± 0.0984	0.7575 ± 0.0519	0.9061 ± 0.0286
MobileNetV3-Large	0.8581 ± 0.0329	0.8466 ± 0.0390	0.7765 ± 0.1115	0.9111 ± 0.0335	0.8053 ± 0.0498	0.9221 ± 0.0253
ResNet-50	0.8521 ± 0.0357	0.8440 ± 0.0726	0.7708 ± 0.1416	0.8952 ± 0.0709	0.7950 ± 0.0632	0.9191 ± 0.0260
ViT-B/16	0.8454 ± 0.0223	0.7794 ± 0.0696	0.8308 ± 0.0767	0.8540 ± 0.0446	0.8018 ± 0.0513	0.9192 ± 0.0286
**Weighted ensemble**	**0.8729 ± 0.0232**	**0.8360 ± 0.0728**	**0.8449 ± 0.1119**	**0.8868 ± 0.0726**	**0.8334 ± 0.0432**	**0.9388 ± 0.0203**

Relative to the strongest single backbone, the weighted ensemble improved mean F1-score by 0.0175 and mean AUC by 0.0104, indicating a modest but consistent gain rather than an abrupt change in operating behaviour. The rank ordering also shows that no single backbone is uniformly best across all metrics: ConvNeXt-Tiny performed best in overall single-model balance, ViT-B/16 favoured recall, and MobileNetV3-Large favoured specificity. Table 7 reports the normalized fold-specific weights selected by the ensemble optimizer. The learned weight distribution did not collapse onto a single dominant backbone; instead, the relative contribution of individual models varied across folds. Averaged across the five folds, the largest weights were assigned to ViT-B/16 (0.252), MobileNetV3-Large (0.208), and ResNet-50 (0.154), whereas ConvNeXt-Tiny, despite being the strongest individual model by mean F1-score, received a more modest average weight (0.106). This pattern suggests that the gain from ensembling arose from complementary error behaviour rather than from simply amplifying the predictions of the best single backbone. The absolute values in Table 6 should be interpreted in light of the stricter protocol used here. Because folds were defined on original images only, augmented images were excluded from reported test evaluation, and thresholds were selected and calibrated within each fold, the present estimates are intentionally more conservative than many single-split or augmentation-heavy reports in the literature. This conservatism is a strength of the benchmark, because it yields performance estimates that are more suitable as reproducible reference values than optimistic headline numbers derived from less restrictive designs.

**Table 7:** Normalized weights selected by random-search optimization for the weighted ensemble across the five cross-validation folds.

Model	Fold 0	Fold 1	Fold 2	Fold 3	Fold 4	Mean
ConvNeXt-Tiny	0.07	0.14	0.10	0.21	0.01	0.106
DenseNet-121	0.03	0.03	0.15	0.03	0.11	0.070
EfficientNet-B0	0.11	0.13	0.23	0.09	0.09	0.130
EfficientNetV2-S	0.09	0.08	0.21	0.02	0.00	0.080
MobileNetV3-Large	0.62	0.09	0.04	0.11	0.18	0.208
ResNet-50	0.05	0.05	0.04	0.16	0.47	0.154
ViT-B/16	0.03	0.48	0.23	0.38	0.14	0.252

### 4.2 Statistical significance of pairwise differences

To assess whether the observed performance differences are attributable to model architecture rather than sampling variability, the four statistical tests described in Section 3.9 were applied to every pairwise model comparison within each fold.

The central finding is that the improvement of the weighted ensemble over the strongest single backbone (ConvNeXt-Tiny) did not reach statistical significance after correction in any individual fold (permutation F1 *p*-values ranged from 0.005 to 0.589; DeLong AUC *p*-values ranged from 0.044 to 0.916). This result is consistent with the modest magnitude of the observed gains (ΔF1  =  0.0175, ΔAUC  =  0.0104) and the limited per-fold test set size (n≈175), and it confirms that the ensemble advantage reported in Table 6 should be interpreted as a consistent directional trend rather than a statistically confirmed improvement.

By contrast, several backbone-level comparisons did reach Bonferroni-corrected significance in at least one fold, confirming that the benchmark retains sufficient statistical power to detect genuine architectural differences when they are large enough. For example, ConvNeXt-Tiny significantly outperformed EfficientNetV2-S in Fold 1 on both McNemar (*p* < 0.001) and permutation F1 (*p* < 0.001), and ViT-B/16 significantly outperformed EfficientNet-B0 in Fold 0 on permutation F1 (*p* < 0.001). The fact that significance was fold-dependent rather than universal further underscores the dataset-driven variability discussed in Section 5 and reinforces the argument that stable claims require larger and more diverse evaluation cohorts.

### 4.3 Model complexity

Table 8 reports the total number of trainable parameters for each backbone after replacing the original classification head with a binary output layer. All backbones used 224×224 input resolution. Parameter counts range from 3.0 M for MobileNetV3-Large to 86.6 M for ViT-B/16, spanning roughly a 29× difference. Despite having the fewest parameters, MobileNetV3-Large achieved the highest single-model specificity (0.9111) and the second-highest F1-score (0.8053), confirming that a lightweight architecture can remain competitive under the present benchmark conditions. Conversely, ViT-B/16, the largest model, yielded the highest single-model recall (0.8308) but not the highest F1-score, indicating that additional model capacity does not uniformly translate into better overall performance when the training set is small. The weighted ensemble aggregates the outputs of all seven backbones and therefore carries the combined parameter budget (approximately 172.1 M) at inference time, which is the principal cost of the ensemble strategy.

**Table 8:** Model complexity of the evaluated backbones (binary classification head, 224×224 input). GFLOPs were computed using the torchprofile library.

Model	Parameters (M)	GFLOPs
MobileNetV3-Large	3.0	0.23
EfficientNet-B0	4.0	0.40
DenseNet-121	7.0	2.87
ResNet-50	23.5	4.12
ConvNeXt-Tiny	27.8	4.47
EfficientNetV2-S	20.2	8.37
ViT-B/16	86.6	17.56
Weighted ensemble (all 7)	∼172.1	∼38.0

### 4.4 Error profile

Table 9 reports the mean confusion-matrix components. The weighted ensemble yielded the highest mean number of true positives (57.8 ± 14.9) and the lowest mean number of false negatives (10.0 ± 6.6), consistent with its leading recall and F1-score. ConvNeXt-Tiny retained a slightly lower false-positive burden (10.8 ± 5.4) than the weighted ensemble while preserving strong AUC. MobileNetV3-Large achieved the lowest mean false-positive count among the single models (9.6 ± 3.8), which aligns with its specificity advantage. These patterns confirm that the benchmark contains clinically relevant operating trade-offs rather than a universally dominant single backbone. Together with Fig 1, the table shows that improvements in one operating characteristic were typically achieved through balanced changes in others rather than through uniformly better performance across every metric.

**Table 9:** Mean confusion-matrix components (mean ± standard deviation) across five folds.

Model	TP	FP	TN	FN
ConvNeXt-Tiny	55.0 ± 15.4	10.8 ± 5.4	96.6 ± 4.5	12.8 ± 5.3
DenseNet-121	54.0 ± 12.9	12.4 ± 5.6	95.0 ± 2.5	13.8 ± 6.1
EfficientNet-B0	51.8 ± 9.9	14.0 ± 4.4	93.4 ± 5.0	16.0 ± 8.3
EfficientNetV2-S	52.4 ± 11.6	17.2 ± 10.7	90.2 ± 12.2	15.4 ± 8.4
MobileNetV3-Large	52.2 ± 9.3	9.6 ± 3.8	97.8 ± 5.4	15.6 ± 9.4
ResNet-50	53.2 ± 16.6	11.4 ± 7.8	96.0 ± 7.0	14.6 ± 6.9
ViT-B/16	56.6 ± 12.7	15.8 ± 5.4	91.6 ± 4.5	11.2 ± 4.8
**Weighted ensemble**	**57.8 ± 14.9**	**12.4 ± 8.4**	**95.0 ± 5.5**	**10.0 ± 6.6**

### 4.5 Calibration quality

To assess the effect of temperature scaling (Section 3.8) on prediction calibration, Table 10 reports the Expected Calibration Error (ECE, 15 equal-width bins) on the held-out test partition of each fold, both before and after temperature scaling. Pre-calibration probabilities were recovered analytically by inverting the per-model temperature parameter (*T*) applied during the original run. Across all five folds, temperature scaling reduced the mean ECE for ViT-B/16 (0.0939 → 0.0815) and for the weighted ensemble (0.1138 → 0.1041), consistent with the known tendency of vision transformers and heterogeneous ensembles to benefit from post hoc calibration. For ConvNeXt-Tiny (0.0768 → 0.0849) and MobileNetV3-Large (0.1034 → 0.1071), the mean ECE increased marginally after scaling; inspection of the per-fold temperatures reveals that these models already had T≈0.5−0.8 in several folds, indicating mild underconfidence that temperature scaling slightly amplified. The overall post-calibration ECE range of 0.05–0.15 indicates moderate calibration quality that is informative for threshold tuning, consistent with the small per-fold test sizes (n≈158−195). Importantly, temperature scaling preserves the monotonic ranking of predicted probabilities even in the cases where bin-wise ECE increases slightly, so the downstream threshold selection step (Section 4.6) still benefits from the scaled outputs.

**Table 10:** Expected Calibration Error (ECE, 15 equal-width bins) before and after temperature scaling, per fold, for the weighted ensemble and the three most informative single backbones.

	Fold 0		Fold 1		Fold 2		Fold 3		Fold 4		Mean	
Model	Pre	Post	Pre	Post	Pre	Post	Pre	Post	Pre	Post	Pre	Post
ConvNeXt-Tiny	0.067	0.067	0.075	0.108	0.093	0.080	0.062	0.058	0.088	0.113	0.077	0.085
ViT-B/16	0.070	0.053	0.103	0.073	0.055	0.057	0.131	0.117	0.110	0.107	0.094	0.081
MobileNetV3-Large	0.133	0.124	0.105	0.149	0.092	0.088	0.090	0.087	0.097	0.087	0.103	0.107
Weighted ensemble	0.126	0.118	0.124	0.149	0.108	0.077	0.111	0.109	0.100	0.067	0.114	0.104

### 4.6 Threshold selection and operating-point verification

Table 11 reports the decision threshold selected on the validation subset of each fold using the f1_at_precision strategy (precision target ≥0.80, minimum recall ≥0.85). For the weighted ensemble, the selected thresholds ranged from 0.323 to 0.485 across folds. The precision target was met on the test partition in 3 of 5 folds (Folds 0, 2, and 4); in Folds 1 and 3 the test precision fell slightly below 0.80 (0.741 and 0.776 respectively), reflecting the difficulty of generalizing a validation-tuned threshold to a small test partition. The recall floor of 0.85 was met in 3 of 5 folds (Folds 1, 2, and 3); the shortfall in Folds 0 and 4 is attributable to the small per-fold positive count (n≈51−63). These values allow readers to reproduce the exact operating point used for each reported confusion matrix and to assess the stability of threshold transfer from validation to test.

**Table 11:** Validation-selected decision thresholds and corresponding test-set precision and recall for the weighted ensemble, per fold.

Fold	Threshold	Test Prec.	Test Rec.	Prec.≥0.80?	Rec.≥0.85?
0	0.389	0.8873	0.7778	✓	×
1	0.323	0.7407	0.9375	×	✓
2	0.365	0.9016	0.8730	✓	✓
3	0.369	0.7755	0.9500	×	✓
4	0.485	0.8750	0.6863	✓	×

### 4.7 Test-time augmentation ablation

To quantify the contribution of 16-fold test-time augmentation (TTA), all models were re-evaluated on the same five test folds with TTA disabled (single-crop inference) using the same saved checkpoints and identical data splits. Table 12 reports the resulting mean F1-score and AUC for the weighted ensemble and the three most informative single backbones. Enabling TTA improved mean F1-score by +0.009 and mean AUC by +0.002 for the weighted ensemble, confirming that TTA provides a consistent but modest gain under the present benchmark conditions. The improvement was largest for MobileNetV3-Large (ΔF1 = +0.026), consistent with the observation that its squeeze-and-excitation blocks benefit from averaging over spatially diverse inputs. ViT-B/16 showed a marginal F1 decrease with TTA (−0.004) despite a slight AUC improvement (+0.002), meaning that the primary ViT-B/16 F1-score reported in Table 6 is 0.0035 lower than it would be under single-crop inference; this suggests that multi-crop averaging occasionally shifts the optimal operating point without improving discrimination for models with global receptive fields. Overall, TTA contributes a small but consistent benefit at the ensemble level, and the moderate absolute improvement confirms that the reported performance is not critically dependent on the TTA protocol.

**Table 12:** Effect of 16-fold test-time augmentation (TTA) on mean F1-score and AUC across five folds.

	TTA off		TTA on (16-fold)	
Model	F1	AUC	F1	AUC
ConvNeXt-Tiny	0.8101	0.9261	0.8159	0.9284
ViT-B/16	0.8053	0.9171	0.8018	0.9192
MobileNetV3-Large	0.7795	0.9182	0.8053	0.9221
Weighted ensemble	0.8246	0.9365	0.8334	0.9388

### 4.8 Fold-specific operating plots

All numerical results reported in this study are based on five-fold cross-validation, not single runs. Tables 6 and 9 report the mean ± standard deviation across the five folds, and Fig 1 displays these fold-level statistics as dot-and-whisker plots in which each point represents the mean and each whisker spans ±1 standard deviation. This visualization confirms that the performance differences between the closest rivals (e.g., the weighted ensemble versus ConvNeXt-Tiny) are accompanied by overlapping standard deviation bands in several metrics, which reflects the genuine difficulty of the task rather than a single-run artefact. The fold-specific confusion matrices, ROC curves, and precision–recall curves shown below are drawn from individual representative folds (Fold 2, Fold 3, and Fold 4 respectively) to illustrate the operating behaviour on a single split; the corresponding aggregate statistics with standard deviations are provided in the tables and in Fig 1.

**Fig 2:**
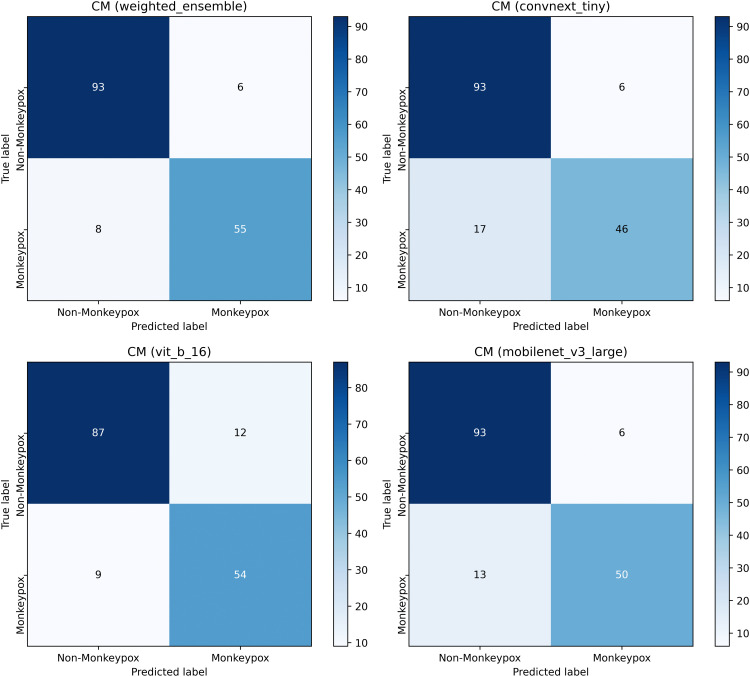
Fold-specific confusion matrices for the weighted ensemble (top-left), ConvNeXt-Tiny (top-right), ViT-B/16 (bottom-left), and MobileNetV3-Large (bottom-right) using Fold 2. These panels visualize the true-positive, false-positive, true-negative, and false-negative trade-offs summarized numerically in Table 9.

**Fig 3:**
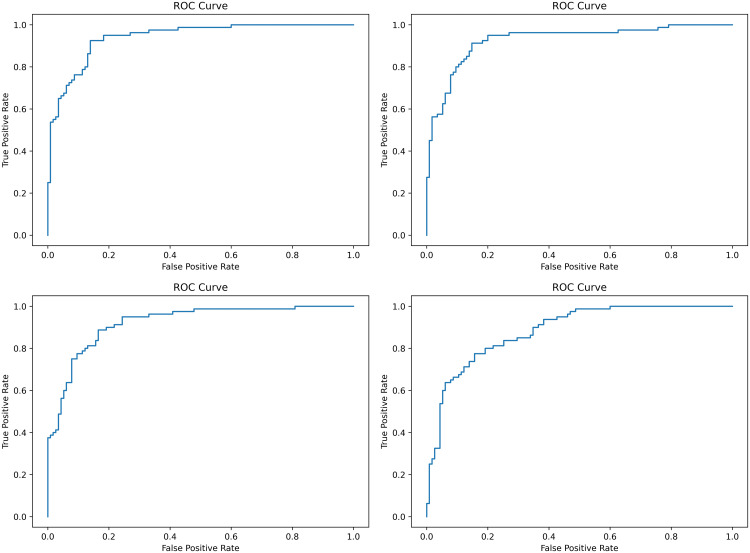
Fold-specific ROC curves for the weighted ensemble (top-left), ConvNeXt-Tiny (top-right), ViT-B/16 (bottom-left), and MobileNetV3-Large (bottom-right) using Fold 3. These panels provide the threshold-varying discrimination view corresponding to the mean AUC values reported in Table 6.

**Fig 4:**
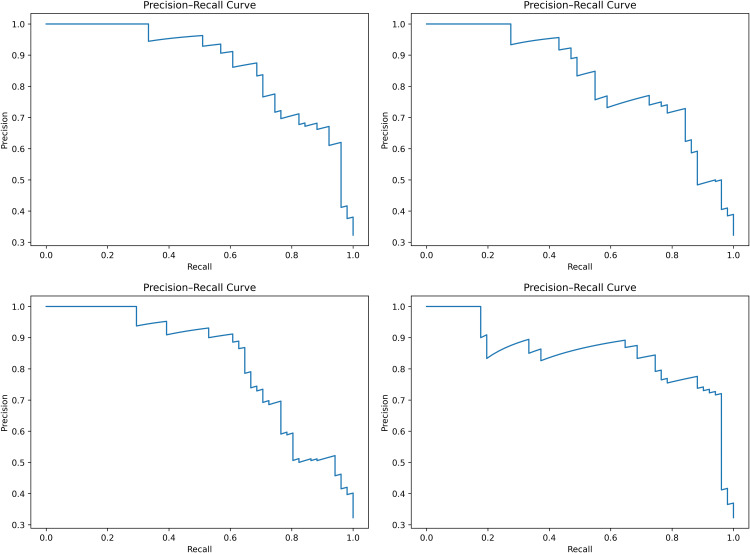
Fold-specific precision–recall curves for the weighted ensemble (top-left), ConvNeXt-Tiny (top-right), ViT-B/16 (bottom-left), and MobileNetV3-Large (bottom-right) using Fold 4. These panels complement the ROC view by emphasizing precision–recall trade-offs under the same benchmark conditions.

To complement the aggregate metrics, Figs 2–4 present fold-specific confusion matrices, receiver operating characteristic (ROC) curves, and precision–recall (PR) curves for the four most informative methods: the weighted ensemble, ConvNeXt-Tiny, ViT-B/16, and MobileNetV3-Large. These methods were selected because they respectively represent the best overall performance, the strongest single-model balance, the strongest recall-oriented single model, and the strongest specificity-oriented single model in Table 6. To avoid overemphasizing any single split, a different representative fold is used for each plot category: **Fold 2** for confusion matrices, **Fold 3** for ROC curves, and **Fold 4** for precision–recall curves. Within each category, all four methods share the same fold so that the visual comparison remains like-for-like. Fig 2 makes the operating trade-offs in Table 9 visually explicit: the weighted ensemble shows the most favorable true-positive/false-negative balance, ConvNeXt-Tiny remains the most balanced single model, ViT-B/16 emphasizes sensitivity, and MobileNetV3-Large limits false positives more aggressively. Fig 3 shows the corresponding ranking in ROC space, where the weighted ensemble provides the strongest overall discrimination and ConvNeXt-Tiny remains the strongest single-model trajectory. Fig 4 provides the complementary precision–recall view, in which the weighted ensemble retains the strongest overall balance while ViT-B/16 and MobileNetV3-Large illustrate recall-oriented and specificity-oriented alternatives.

## 5 Discussion

The benchmark demonstrates that strong binary mpox classification can be obtained from a unified curated lesion-image dataset, but it also shows that performance remains meaningfully imperfect under a stricter protocol built around group-aware splitting, original-only test evaluation, calibrated thresholds, and fold-wise reporting. This distinction matters because public lesion-image datasets often contain repeated variants, heterogeneous acquisition conditions, and mixed source provenance, all of which can inflate apparent performance if evaluation design is not carefully controlled [9]. In this sense, the comparatively moderate results reported here should not be read as a weakness relative to earlier, higher literature figures; rather, they reflect an intentional shift toward a more conservative and auditable evaluation design.

The relative ranking of the evaluated methods is consistent with this interpretation. The weighted ensemble produced the best overall F1-score and AUC, suggesting that combining calibrated model outputs is beneficial when source heterogeneity and class overlap remain present. However, the gain over the best single backbone was moderate rather than dramatic, and fold-level statistical testing (Section 4.2) confirmed that this difference did not reach significance after Bonferroni correction, which indicates that the ensemble improves robustness without removing the underlying difficulty of the task. Among the individual backbones, ConvNeXt-Tiny provided the best overall balance, ViT-B/16 was more recall-oriented, and MobileNetV3-Large was more specificity-oriented. This pattern is practically useful because the preferred operating point depends on the intended deployment context. A screening-oriented workflow may prioritize recall and low false negatives, whereas a triage setting seeking to limit unnecessary referrals may place more emphasis on specificity.

It is important to note that the fold-level variability in several metrics is substantial and should be interpreted as an informative constraint rather than a limitation of the modelling alone. For instance, the weighted ensemble recall standard deviation of 0.1119 implies that individual-fold recall values plausibly range from approximately 0.73 to 0.96, meaning that in the least favourable fold roughly one in four mpox cases would be missed. Similarly, ResNet-50 exhibited a recall standard deviation of 0.1416, the highest among all evaluated models. These fluctuations are primarily attributable to the small per-fold test sets (approximately 68 positive and 107 negative images per fold) and to source-level heterogeneity in the underlying public data rather than to architectural deficiency. The fold-wise ensemble weight distribution in Table 7 corroborates this interpretation: the dominant backbone shifts across folds (for example, MobileNetV3-Large receives weight 0.62 in Fold 0 but only 0.04 in Fold 2), indicating that the difficulty of each test partition varies enough to alter the optimal model mixture. These patterns define a performance plateau at which further architectural tuning is unlikely to yield stable gains without larger, more diverse, and externally validated image collections.

Several architectural and data-level factors may explain the observed performance patterns. First, ConvNeXt-Tiny achieved the strongest single-model F1-score, consistent with the hypothesis that its hierarchical convolutional design with depthwise convolutions and large 7×7 kernels captures both local texture cues (e.g., vesicle boundaries and pustule morphology) and medium-range spatial context, which are likely among the primary discriminative features in lesion imagery. Second, ViT-B/16 exhibited the highest recall, plausibly reflecting its global self-attention mechanism that distributes receptive-field coverage uniformly across the image from the first layer, making it less likely to miss atypical or peripherally located lesions; however, this same property may increase false-positive susceptibility when background skin regions share colour statistics with early-stage lesions, which would account for its comparatively lower precision. Third, MobileNetV3-Large achieved the highest specificity despite having the fewest parameters (3.0 M), which is consistent with the channel-wise recalibration performed by its squeeze-and-excitation blocks that may effectively suppress non-lesion texture responses, yielding conservative positive predictions at the cost of lower recall. Fourth, the weighted ensemble outperformed all single backbones, which is consistent with the expectation that architecturally diverse error profiles (convolutional locality bias in ConvNeXt-Tiny and MobileNetV3, global attention bias in ViT-B/16, and residual gradient flow differences in ResNet-50 and DenseNet-121) are sufficiently complementary that a learned convex combination reduces the variance of the final prediction without requiring additional training data. Finally, the moderate absolute performance level (F1 ≈ 0.83 rather than >0.95) is best explained by the data characteristics themselves: the unified benchmark merges two independently collected sources with different acquisition devices, lighting conditions, and racial diversity profiles, and the strict exclusion of augmented images from test evaluation removes the artificial variance reduction that augmentation-based protocols provide. Under these conditions, the performance ceiling is constrained by inter-source heterogeneity and the relatively small number of original mpox images (339), rather than by any single modelling choice.

The present results fit within the broader mpox literature but should not be reduced to a leaderboard comparison. As summarized in Table 1, published peer-reviewed studies report accuracies ranging from approximately 87% under conservative protocols [5,13] to above 99% under augmentation-heavy or validation-only designs [17,19,21]. Three factors account for most of this spread. First, class granularity varies: binary mpox-versus-others settings tend to yield higher headline figures than multiclass differential-diagnosis settings [11,14]. Second, augmentation intensity differs markedly; studies that report test metrics on augmented data consistently outperform those that restrict evaluation to original images [11,12]. Third, evaluation design ranges from single predefined splits with no leakage analysis [6,17] to cross-validated but non-grouped schemes [14], and the ranking of backbones has been shown to change between datasets [7]. The present benchmark’s accuracy of 0.8729 and F1-score of 0.8334 under grouped original-only evaluation therefore falls at the conservative end of the published range, which is consistent with the deliberately strict protocol rather than indicative of modelling weakness. In this context, the more informative comparison is not with the absolute peak figures in the literature but with the degree to which the present design controls the confounds that inflate those figures.

Several limitations should be acknowledged. First, the unified benchmark relies on public lesion images rather than prospectively collected clinical data; external validation on an independent clinical cohort remains essential before the reported performance can be interpreted as deployment-ready. To mitigate this limitation, the study incorporates a rigorous suite of internal robustness analyses that collectively provide stronger evidence of methodological soundness than is typical of single-dataset benchmarks: (i) content-level near-duplicate auditing via perceptual hashing confirmed zero cross-fold leaks across 876 images (Section 3.3); (ii) pre- and post-calibration ECE was quantified for every model in every fold, revealing both the benefits and limits of temperature scaling (Section 4.5); (iii) per-fold decision thresholds were reported with explicit verification of whether precision and recall targets were met on the test partition (Section 4.6); (iv) a TTA ablation using the same saved checkpoints and identical data splits demonstrated that the reported results are not critically dependent on the test-time augmentation protocol (Section 4.7); and (v) four complementary statistical tests with Bonferroni correction were applied to all pairwise model comparisons within each fold (Section 4.2). While these analyses do not substitute for external validation, they ensure that the reported performance estimates are internally consistent, auditable, and not inflated by methodological artefacts.

Second, although the perceptual-hash audit confirmed that no content-level near-duplicate leaked across folds, these checks cannot detect semantically similar images that differ at the pixel level (e.g., the same lesion photographed from slightly different angles); true patient-level identity control would require metadata that the public MSLD sources do not provide. Third, the benchmark is binary, whereas real clinical workflows often require richer differential diagnosis across multiple dermatological conditions and acquisition settings. Although MSLD v2.0 was developed with explicit attention to racial diversity, the present benchmark does not include image-level demographic or skin-tone annotations; therefore, fairness across skin tones cannot be directly assessed and remains an important direction for future work [22].

These limitations are consistent with the critique summarized by Hossain et al., who highlighted dataset quality, weak generalizability, lesion variability, image noise, and inconsistent benchmarking as open problems in this field [9]. The present benchmark addresses part of that problem by specifying how data were selected, grouped, mixed, thresholded, calibrated, and reported, and by making the resulting estimates deliberately conservative. Future studies should build on this by introducing external validation cohorts, explicit patient-level identifiers, richer clinical metadata, and prospective evaluation in realistic acquisition settings.

## 6 Conclusion

This study presented a leakage-aware benchmark for binary mpox skin-image classification using a unified curated dataset assembled from MSLD v1.0 and MSLD v2.0. Seven pretrained backbones and a weighted ensemble were compared under a shared protocol defined by group-stratified 5-fold cross-validation, original-only test evaluation, controlled use of augmented images during training, original-only fine-tuning, validation-based threshold selection, and temperature scaling.

The weighted ensemble achieved the best overall performance, with mean accuracy of 0.8729 ± 0.0232, precision of 0.8360 ± 0.0728, recall of 0.8449 ± 0.1119, specificity of 0.8868 ± 0.0726, F1-score of 0.8334 ± 0.0432, and AUC of 0.9388 ± 0.0203 across the five folds. Among the individual backbones, ConvNeXt-Tiny provided the strongest overall single-model performance (F1-score 0.8159 ± 0.0727). The results show that weighted ensembling improves robustness under a carefully controlled evaluation setting, but the substantial fold-level variability, particularly in recall (SD = 0.1119 for the ensemble), confirms that performance remains dataset-dependent and that the current public data do not yet support stable, deployment-grade classification.

The main contribution of this work is therefore methodological: a transparent benchmark in which dataset construction, group handling, training policy, threshold selection, calibration, and evaluation protocol are documented in sufficient detail to support meaningful interpretation. In relation to the existing mpox imaging literature, the study contributes a stricter and more reproducible reference design rather than a new architecture, which is why its value lies in the credibility of the benchmark rather than in a single peak accuracy claim. Future work should extend this line of research through externally validated datasets, explicit patient-level deduplication, richer multiclass differential diagnosis, and prospective real-world assessment.

## References

[1] Bryant AE, Shulman ST. Mpox: emergence following smallpox eradication, ongoing outbreaks and strategies for prevention. Curr Opin Infect Dis. 2025;38(3):222–7. doi: 10.1097/QCO.0000000000001100 39878084

[2] Altindis M, Puca E, Shapo L. Diagnosis of monkeypox virus - An overview. Travel Med Infect Dis. 2022;50:102459. doi: 10.1016/j.tmaid.2022.102459 36109000 PMC9534096

[3] Khan A, Aziz S, Fayaz M, Shah B, Algarni F. A CNN-LSTM model for sentiment analysis on monkeypox tweets. New Generation Computing. 2023;42:89–107. doi: 10.1007/s00354-023-00227-0

[4] Chadaga K, Prabhu S, Sampathila N, Nireshwalya S, Katta SS, Tan RS, et al. Application of Artificial Intelligence Techniques for Monkeypox: A Systematic Review. Diagnostics. 2023;13(5):824. doi: 10.3390/diagnostics13050824 36899968 PMC10000611

[5] Sitaula C, Shahi TB. Monkeypox Virus Detection Using Pre-trained Deep Learning-based Approaches. J Med Syst. 2022;46(11):78. doi: 10.1007/s10916-022-01868-2 36201085 PMC9535233

[6] Sahin VH, Oztel I, Yolcu Oztel G. Human Monkeypox Classification from Skin Lesion Images with Deep Pre-trained Network using Mobile Application. J Med Syst. 2022;46(11):79. doi: 10.1007/s10916-022-01863-7 36210365 PMC9548428

[7] Almufareh MF, Tehsin S, Humayun M, Kausar S. A Transfer Learning Approach for Clinical Detection Support of Monkeypox Skin Lesions. Diagnostics (Basel). 2023;13(8):1503. doi: 10.3390/diagnostics13081503 37189603 PMC10137438

[8] Muñoz-Saavedra L, Escobar-Linero E, Civit-Masot J, Luna-Perejón F, Civit A, Domínguez-Morales M. A Robust Ensemble of Convolutional Neural Networks for the Detection of Monkeypox Disease from Skin Images. Sensors (Basel). 2023;23(16):7134. doi: 10.3390/s23167134 37631672 PMC10459252

[9] Hossain MS, Ahmed M, Rahman MS. From survey to solution: A deep learning framework for reliable monkeypox diagnosis using skin images. Array. 2025;28:100554. doi: 10.1016/j.array.2025.100554

[10] Vega C, Schneider R, Satagopam V. Analysis: Flawed Datasets of Monkeypox Skin Images. J Med Syst. 2023;47(1):37. doi: 10.1007/s10916-023-01928-1 36933065 PMC10024024

[11] Bala D, Hossain MS, Hossain MA, Abdullah MI, Rahman MM, Manavalan B, et al. MonkeyNet: A robust deep convolutional neural network for monkeypox disease detection and classification. Neural Netw. 2023;161:757–75. doi: 10.1016/j.neunet.2023.02.022 36848828 PMC9943560

[12] Altun M, Gürüler H, Özkaraca O, Khan F, Khan J, Lee Y. Monkeypox Detection Using CNN with Transfer Learning. Sensors (Basel). 2023;23(4):1783. doi: 10.3390/s23041783 36850381 PMC9964526

[13] Uysal F. Detection of Monkeypox Disease from Human Skin Images with a Hybrid Deep Learning Model. Diagnostics (Basel). 2023;13(10):1772. doi: 10.3390/diagnostics13101772 37238256 PMC10217161

[14] Sorayaie Azar A, Naemi A, Babaei Rikan S, Bagherzadeh Mohasefi J, Pirnejad H, Wiil UK. Monkeypox detection using deep neural networks. BMC Infect Dis. 2023;23(1):438. doi: 10.1186/s12879-023-08408-4 37370031 PMC10304329

[15] Thieme AH, Zheng Y, Machiraju G, Sadee C, Mittermaier M, Gertler M, et al. A deep-learning algorithm to classify skin lesions from mpox virus infection. Nat Med. 2023;29(3):738–47. doi: 10.1038/s41591-023-02225-7 36864252 PMC10033450

[16] Nayak T, Chadaga K, Sampathila N, Mayrose H, Muralidhar Bairy G, Prabhu S. Detection of monkeypox from skin lesion images using deep learning networks and explainable artificial intelligence. Applied Mathematics in Science and Engineering. 2023;31(1):2225698. doi: 10.1080/27690911.2023.2225698

[17] Nayak T, Chadaga K, Sampathila N, Mayrose H, Gokulkrishnan N, Bairy MG, et al. Deep learning based detection of monkeypox virus using skin lesion images. Medicine in Novel Technology and Devices. 2023;18:100243. doi: 10.1016/j.medntd.2023.100243 37293134 PMC10236906

[18] Pramanik R, Banerjee B, Efimenko G, Kaplun D, Sarkar R. Monkeypox detection from skin lesion images using an amalgamation of CNN models aided with Beta function-based normalization scheme. PLoS One. 2023;18(4):e0281815. doi: 10.1371/journal.pone.0281815 37027356 PMC10081766

[19] Bamaqa A, Bahgat WM, AbdulAzeem Y, Balaha HM, Badawy M, Elhosseini MA. Early detection of monkeypox: Analysis and optimization of pretrained deep learning models using the Sparrow Search Algorithm. Results in Engineering. 2024;24:102985. doi: 10.1016/j.rineng.2024.102985

[20] Aslam S, Khan MU, Arif S, Khosa SA. Monkeypox recognition and prediction from visuals using deep transfer learning-based neural networks. Multimedia Tools and Applications. 2024;83:67543–68. doi: 10.1007/s11042-024-18437-z

[21] Elhadidy MS, Elgohr AT, Mousa A, Safwat A, Abdelfatah RI, Kasem HM. Benchmarking Pre-trained CNNs and Vision Transformers for Mpox-related Dermatological Image Classification on MSLD v2.0. Results in Engineering. 2025;28:108071. doi: 10.1016/j.rineng.2025.108071

[22] Ali SN, Ahmed MdT, Jahan T, Paul J, Sakeef Sani SM, Noor N, et al. A web-based mpox skin lesion detection system using state-of-the-art deep learning models considering racial diversity. Biomedical Signal Processing and Control. 2024;98:106742. doi: 10.1016/j.bspc.2024.106742

[23] Shorten C, Khoshgoftaar TM. A survey on Image Data Augmentation for Deep Learning. J Big Data. 2019;6(1). doi: 10.1186/s40537-019-0197-0 PMC8287113 34306963

[24] Zauner C. Implementation and Benchmarking of Perceptual Image Hash Functions [Diploma Thesis]. Upper Austria University of Applied Sciences: Hagenberg Campus. 2010.

[25] Tan M, Le QV. In: Proceedings of the 38th International Conference on Machine Learning (ICML), 2021. 10096–106. 10.48550/arXiv.2104.00298

[26] Liu Z, Mao H, Wu C-Y, Feichtenhofer C, Darrell T, Xie S. A ConvNet for the 2020s. In: 2022 IEEE/CVF Conference on Computer Vision and Pattern Recognition (CVPR), 2022. 11966–76. 10.1109/cvpr52688.2022.01167

[27] Dosovitskiy A, Beyer L, Kolesnikov A, Weissenborn D, Zhai X, Unterthiner T, et al. An Image is Worth 16x16 Words: Transformers for Image Recognition at Scale. In: 2021. 10.48550/arXiv.2010.11929

[28] He K, Zhang X, Ren S, Sun J. Deep Residual Learning for Image Recognition. In: 2016 IEEE Conference on Computer Vision and Pattern Recognition (CVPR), 2016. 770–8. 10.1109/cvpr.2016.90

[29] Huang G, Liu Z, Van Der Maaten L, Weinberger KQ. Densely Connected Convolutional Networks. In: 2017 IEEE Conference on Computer Vision and Pattern Recognition (CVPR), 2017. 2261–9. 10.1109/cvpr.2017.243

[30] Tan M, Le QV. In: Proceedings of the 36th International Conference on Machine Learning (ICML), 2019. 6105–14. 10.48550/arXiv.1905.11946

[31] Howard A, Sandler M, Chen B, Wang W, Chen L-C, Tan M, et al. Searching for MobileNetV3. In: 2019 IEEE/CVF International Conference on Computer Vision (ICCV), 2019. 1314–24. 10.1109/iccv.2019.00140

[32] Guo C, Pleiss G, Sun Y, Weinberger KQ. In: Proceedings of the 34th International Conference on Machine Learning (ICML), 2017. 1321–30. 10.48550/arXiv.1706.04599

[33] Akiba T, Sano S, Yanase T, Ohta T, Koyama M. In: Proceedings of the 25th ACM SIGKDD International Conference on Knowledge Discovery and Data Mining (KDD), 2019. 2623–31. 10.1145/3292500.3330701

[34] Fawcett T. An introduction to ROC analysis. Pattern Recognition Letters. 2006;27(8):861–74. doi: 10.1016/j.patrec.2005.10.010

[35] McNemar Q. Note on the sampling error of the difference between correlated proportions or percentages. Psychometrika. 1947;12(2):153–7. doi: 10.1007/BF02295996 20254758

[36] Wilcoxon F. Individual comparisons by ranking methods. Biometrics Bulletin. 1945;1(6):80–3. doi: 10.2307/3001968

[37] DeLong ER, DeLong DM, Clarke-Pearson DL. Comparing the areas under two or more correlated receiver operating characteristic curves: a nonparametric approach. Biometrics. 1988;44(3):837–45. doi: 10.2307/2531595 3203132

