# Correction: AI-driven diagnosis of mpox using deep learning models

**DOI:** 10.1371/journal.pone.0357001

**Published:** 2026-08-25

**Authors:** Bassam W. Aboshosha, Shafiq Ul Rehman, Lamees N. Mahmoud, Ibrahim Sadek

There are errors in the author affiliations. The correct affiliations are as follows:

Bassam W. Aboshosha^1^, Shafiq Ul Rehman^2^, Lamees N. Mahmoud^3^, Ibrahim Sadek^3^

**1** School of Engineering and Computer Science, University of Hertfordshire, Hosted by Global Academic Foundation, New Administrative Capital, Egypt, **2** College of Information Technology, Kingdom University, Riffa, Kingdom of Bahrain, **3** Biomedical Engineering Department, Faculty of Engineering, Capital University (formerly Helwan University), Helwan, Egypt.
