# Supplementary material for: AI-driven diagnosis of mpox using deep learning models
Source: PLoS One. 2026 Jul 10;21(7):e0352161. doi: 10.1371/journal.pone.0352161 (PMC13354086; doi:10.1371/journal.pone.0352161)
Supplement: Attachment — Submitted filename: response_to_reviewers.docx [file pone.0352161.s001.docx]

POINT-BY-POINT RESPONSE TO EDITOR AND REVIEWER COMMENTS

=========================================================

Manuscript ID: PONE-D-25-66974

Manuscript Title: AI-Driven Diagnosis of Monkeypox using Deep Learning Models

We sincerely thank the Associate Editor, Dr. Morufu Olalekan Raimi, and the two Reviewers for their thorough and constructive evaluation. Every concern has been carefully considered and has led to a substantially revised manuscript. Below we provide a detailed point-by-point response. All referenced sections, tables, and figures refer to the revised manuscript.

=========================================================

RESPONSE TO ASSOCIATE EDITOR

=========================================================

EDITOR CONCERN 1: Insufficient Methodological Contribution

-----------------------------------------------------------

"There is no novelty in the methodology. This study is totally relied on established architectures without any significant advancement."

RESPONSE:

We fully agree that the former manuscript did not clearly articulate its contribution and read as a replication study. The revised manuscript has been completely restructured. The contribution is now explicitly framed as methodological rather than architectural. The study does not propose a new backbone; instead, it provides a unified curation recipe, a leakage-aware validation design, and fold-wise reference results intended to support defensible comparison with prior and future mpox skin-image studies.

Specifically, the revised Introduction (Section 1) states three objectives: (1) construct a unified curated binary dataset with fully specified class-mapping and selection rules; (2) compare multiple pretrained models and a weighted ensemble under a shared pipeline; and (3) examine how dataset construction and leakage-aware evaluation influence the interpretation of performance on public mpox lesion data. Three research questions (RQ1-RQ3) are enumerated to precisely define the scope.

This repositioning addresses the novelty concern by delineating what the study contributes (a rigorous, auditable benchmark design) from what it does not claim (a new architecture).

EDITOR CONCERN 2: Literature Review Incomplete, Contribution Unclear

---------------------------------------------------------------------

"The literature review doesn't provide comprehensive coverage of related work. Most troublesome is the significant gap in coverage of the state-of-the-art."

RESPONSE:

Section 2 (Related work) has been entirely rewritten. It now includes a narrative review of 14 peer-reviewed studies spanning 2022-2025, covering transfer learning, explainable AI, ensemble methods, mobile deployment, and optimization-based approaches. A new standardized comparison table (Table 1) presents 12 representative studies with five columns: Study, Task/dataset, Split/validation, Best reported result, and Comparability limitation. Each row explicitly explains why direct comparison with the present benchmark is limited, preventing misleading leaderboard-style comparisons.

The research gap is now clearly identified in the Introduction: heterogeneous benchmarking practices prevent meaningful comparison across studies. This is supported by recent critiques from Hossain et al. (2025), who highlighted dataset quality and inconsistent benchmarking as major barriers, and Vega et al. (2023), who showed that some early mpox datasets contained flawed or medically irrelevant web-scraped content.

The following studies have been added to strengthen the coverage:

- Thieme et al. (2023, Nature Medicine): deep-learning classification of mpox skin lesions with geographically diverse clinical validation

- Pramanik et al. (2023, PLOS ONE): CNN amalgamation with Beta function-based normalization

- Aslam et al. (2024): deep transfer-learning approach for monkeypox visual recognition (as requested by Reviewer 2)

- Khan et al. (2023): CNN-LSTM sentiment analysis on monkeypox tweets (as requested by Reviewer 2)

- Elhadidy et al. (2025): multiclass benchmarking on MSLD v2.0

EDITOR CONCERN 3: Dataset Description Inadequate

-------------------------------------------------

"There is no such sub-section mentioning the details about the dataset size. I couldn't find anything regarding the size or quantity of the dataset."

RESPONSE:

Section 3.2 (Unified dataset construction) now provides complete documentation:

- Sources: MSLD v1.0 (cited via Almufareh et al. 2023) and MSLD v2.0 (cited via Ali et al. 2024), both publicly available repositories.

- Size: 876 original images (339 mpox, 537 non-mpox) and 1,357 total images including augmented variants.

- Class distribution: 38.7% mpox / 61.3% non-mpox. The majority-class baseline accuracy (ZeroR = 0.613) is reported in the Results section to contextualize model performance.

- Input resolution: All images resized to 224 x 224 pixels.

- Normalization: ImageNet mean and standard deviation.

- Class mapping: Explicit rules for how MSLD v1.0 and v2.0 categories were mapped to the binary mpox/non-mpox labels.

Section 3.3 (Leakage control, grouping, and split policy) provides:

- Grouping: 617 filename-derived groups using a specified regular expression, with an average of 1.42 images per group and a maximum of 14.

- Near-duplicate audit: A 64-bit perceptual-hash (pHash) audit was conducted over all 876 original images. The audit identified 95 candidate near-duplicate pairs (Hamming distance <= 10); in every case, both members belonged to the same cross-validation fold, yielding zero cross-fold leaks.

- Augmentation control: Augmented images capped at 3 variants per group (citing Shorten and Khoshgoftaar 2019); original-image training ratio set to 0.70.

- Potential confounders: The Discussion acknowledges that source-level heterogeneity (different acquisition devices, lighting conditions, and racial diversity profiles between MSLD v1.0 and v2.0) is a primary driver of fold-level variability.

EDITOR CONCERN 4: Experimental Details Insufficient for Reproducibility

------------------------------------------------------------------------

"Critical missing information includes: preprocessing details, augmentation details, training details, fine-tuning details, hardware/software, hyperparameter selection."

RESPONSE:

All requested experimental details are now fully documented:

- Preprocessing: Images resized to 224 x 224, normalized with ImageNet mean [0.485, 0.456, 0.406] and standard deviation [0.229, 0.224, 0.225]. Augmented images excluded from fold construction and from all test/validation evaluations.

- Augmentation: Augmented variants used only during training, capped at 3 per group. Online augmentation parameters (MixUp alpha, CutMix alpha, RandAugment) are reported per backbone in Table 3.

- Training: Optimizer, learning rate, weight decay, label smoothing, loss function (cross-entropy or focal), scheduler, warmup epochs, gradient clipping, and drop-path rate are reported per backbone in Tables 2-3. These were selected by an NSGA-II multi-objective hyperparameter search (840 trials, 6 epochs each).

- Fine-tuning: After main training, a 5-epoch fine-tuning phase at lr = 1e-5 was applied on original-only training data to address the augmented-to-original domain shift. All layers were unfrozen. MixUp and CutMix were disabled during fine-tuning.

- Early stopping: Patience = 4 epochs, monitored metric = f1_at_precision on the validation set.

- Hardware: NVIDIA A100 (40 GB) GPU for training; NVIDIA GeForce RTX 4060 Laptop GPU for TTA ablation re-evaluation (Section 3.9).

- Software: Python 3.10, PyTorch 2.1, torchvision 0.16, Optuna 3.4, scikit-learn 1.3, SciPy 1.11, ImageHash.

- Seeds: 12345-12349 (one per fold), fixed for reproducibility.

- Compute budget: 15-25 GPU-hours total.

- Pretrained weights: torchvision ImageNet-1K V1 variants for all backbones (Section 3.9).

- Model complexity: Trainable parameters and GFLOPs (computed via the torchprofile library) are reported in Table 4.

EDITOR CONCERN 5: Near-Perfect Performance Raises Concerns

-----------------------------------------------------------

"EfficientNet achieving 99.64% validation accuracy with 100% precision and recall on a medical image classification task is extraordinarily high."

RESPONSE:

This concern has been fully resolved. The former manuscript's near-perfect results have been replaced with substantially lower and intentionally conservative results obtained under a stricter protocol:

- Weighted ensemble: F1 = 0.8334 +/- 0.0432, AUC = 0.9388 +/- 0.0203

- Best single model (ConvNeXt-Tiny): F1 = 0.8159 +/- 0.0727, AUC = 0.9284 +/- 0.0303

These results are reported under 5-fold group-stratified cross-validation with original-only test evaluation, meaning no augmented images appear in test or validation sets. The abstract explicitly characterizes these as "deflated but more trustworthy reference values."

Five robustness analyses substantiate the integrity of these results:

(i) A perceptual-hash near-duplicate audit confirmed zero cross-fold data leakage across all 876 images (Section 3.3).

(ii) Pre- and post-calibration Expected Calibration Error (ECE) is quantified for every model in every fold, revealing both the benefits and limits of temperature scaling (Section 4.6, Table 5).

(iii) Per-fold decision thresholds are reported with explicit verification of whether precision (>= 0.80) and recall (>= 0.85) targets were met on the test partition (Section 4.7, Table 7).

(iv) A TTA ablation using the same saved checkpoints and identical data splits demonstrates that the reported results are not critically dependent on the test-time augmentation protocol; the ensemble F1 gain from TTA is +0.009 (Section 4.8, Table 8).

(v) Four complementary statistical tests (McNemar, permutation, Wilcoxon signed-rank, DeLong) with Bonferroni correction are applied to all pairwise model comparisons (Section 4.5).

Error analysis is provided through fold-wise confusion matrix components (Table 8, showing TP, FP, TN, FN per fold) and fold-specific confusion matrix, ROC, and precision-recall plots (Figures 1-4).

EDITOR CONCERN 6: Smart Clinic Concept Underdeveloped

------------------------------------------------------

"The concept of an unmanned smart clinic is underdeveloped. It is not integrated with the core analysis."

RESPONSE:

The smart clinic concept has been entirely removed from the revised manuscript. The paper is now purely a benchmarking study focused on methodological rigour in mpox skin-image classification. This decision was made because the clinic concept was a conceptual proposal without empirical validation, as the Editor correctly noted.

EDITOR CONCERN 7: Literature Comparison Table Problematic

----------------------------------------------------------

"The table does not indicate dataset sizes, class distributions, or validation methodologies, making comparisons meaningless."

RESPONSE:

Table 1 has been completely redesigned as a standardized longtable with five columns: Study, Task/dataset, Split/validation, Best reported result, and Comparability limitation. It now covers 12 peer-reviewed studies spanning 2022-2025. Each entry documents the dataset size, class structure, validation strategy, and best reported result, alongside a specific explanation of why direct comparison with the present benchmark is limited (e.g., "binary evaluation performed on an augmented four-class pool," "headline accuracy is a validation figure obtained during hyperparameter search," "model ranking reverses across datasets"). The present study is included as the final row with a gray highlight. This design ensures that readers can assess comparability rather than simply rank headline numbers.

EDITOR CONCERN 8: Writing and Presentation

-------------------------------------------

"The manuscript has significant presentation problems: redundancy, organization, figures, table formatting, references."

RESPONSE:

The manuscript has been completely rewritten and restructured:

- Structure: Introduction, Related work, Materials and methods (with 9 subsections), Results (with 9 subsections including new calibration, threshold, and TTA analyses), Discussion, Conclusion. No redundancy between sections.

- Figures: All figures are in PNG format with self-explanatory captions that expand all acronyms and explain color/linestyle legends. Confusion matrices, ROC curves, and precision-recall curves are presented for four representative models using different folds to avoid overemphasizing any single split.

- Tables: 9 tables, all using booktabs formatting (toprule, midrule, bottomrule) with no vertical rules. Wide tables use resizebox to prevent overflow. Statistical notation (mean +/- SD) is consistent throughout.

- References: 38 references, all integrated into the narrative with specific attribution. PLOS ONE Vancouver style (plos2025.bst). All entries include DOIs.

- Formatting: Line numbers and double spacing enabled. 10pt font, letterpaper, 1-inch margins.

EDITOR REQUIRED REVISION E: External Validation

-------------------------------------------------

"Test the models on an independent dataset not used in development. If external validation is not possible, explicitly discuss this as a major limitation."

RESPONSE:

External validation on an independent clinical cohort was not performed in this revision. While other public mpox-related image collections exist, these differ substantially from the present benchmark in class definitions, labeling protocols, image quality, and acquisition conditions. Using them as an external test set without careful harmonization would introduce confounds that undermine the interpretability of the comparison — precisely the problem this benchmark is designed to address. Rather than report a potentially misleading external validation on incompatible data, we chose to strengthen the internal evidence base through a comprehensive suite of robustness analyses and to identify external validation on a prospectively collected, protocol-matched clinical cohort as the highest priority for future work. This limitation is explicitly acknowledged in the Discussion as the first limitation listed. However, the revised manuscript compensates for this gap with a comprehensive suite of internal robustness analyses that collectively provide stronger evidence of methodological soundness than is typical of single-dataset benchmarks:

(i) Content-level near-duplicate auditing via perceptual hashing confirmed zero cross-fold leaks across 876 images (Section 3.3).

(ii) Pre- and post-calibration ECE was quantified for every model in every fold, revealing both the benefits and limits of temperature scaling (Section 4.6).

(iii) Per-fold decision thresholds were reported with explicit verification of whether precision and recall targets were met on the test partition (Section 4.7).

(iv) A TTA ablation using the same saved checkpoints and identical data splits demonstrated that the reported results are not critically dependent on the test-time augmentation protocol (Section 4.8).

(v) Four complementary statistical tests with Bonferroni correction were applied to all pairwise model comparisons within each fold (Section 4.5).

While these analyses do not substitute for external validation, they ensure that the reported performance estimates are internally consistent, auditable, and not inflated by methodological artefacts. External validation on a prospectively collected clinical cohort is identified as the highest priority for future work in both the Limitations paragraph and the Conclusion.

=========================================================

RESPONSE TO REVIEWER 1

=========================================================

REVIEWER 1: "There is no novelty in the methodology. This study is totally relied on established architectures without any significant advancement. The paper is not suitable for a reputable journal like plos one."

RESPONSE:

We respectfully acknowledge this concern and agree that the former manuscript failed to articulate a clear contribution. The revised manuscript has been completely reframed. The contribution is now explicitly methodological: a transparent benchmark protocol that documents dataset construction, group handling, leakage controls, calibration, threshold selection, and fold-wise evaluation in sufficient detail to support reproducible comparison.

The revised Introduction states three objectives and three research questions (RQ1-RQ3). The title, abstract, introduction, discussion, and conclusion all consistently frame the study as a benchmark contribution rather than an architectural one. Specifically, the Introduction states: "The contribution is therefore methodological rather than architectural: the study does not introduce a new backbone, but instead provides a unified curation recipe, a leakage-aware validation design, and fold-wise reference results intended to support more defensible comparison with prior and future mpox skin-image studies."

Additionally, the revised manuscript now includes several substantive analytical contributions that were absent from the former version: a perceptual-hash near-duplicate audit, pre/post-calibration ECE analysis, per-fold threshold verification, a TTA ablation, and four-way statistical testing with Bonferroni correction. These go beyond what is typically reported in mpox imaging studies and constitute a meaningful methodological advance in benchmarking practice for this domain.

Regarding technical soundness and data availability (where Reviewer 1 answered "No" to both): the revised manuscript now reports 5-fold group-stratified cross-validation with mean +/- SD for all metrics, four statistical tests with multiple-comparisons correction, and all data and code are publicly available via GitHub (https://github.com/ibrahimsadek/mpox_monkey). The manuscript has been professionally rewritten in standard English.

=========================================================

RESPONSE TO REVIEWER 2

=========================================================

REVIEWER 2, POINT 1: Objectives and Research Questions

-------------------------------------------------------

"Please add two paragraphs in the introduction: a) objectives and motivations tied to gaps in the literature; b) research questions."

RESPONSE:

The Introduction now includes both requested elements. Three objectives are stated: (1) construct a unified curated binary dataset with fully specified class-mapping rules; (2) compare multiple pretrained models and an ensemble under a shared pipeline; (3) examine how dataset construction and leakage-aware evaluation influence performance interpretation. Three research questions follow:

- RQ1: Under a shared group-aware evaluation protocol, which pretrained backbone provides the most reliable performance for binary mpox image classification?

- RQ2: Does weighted ensembling provide a consistent advantage over individual backbones when testing is restricted to original images and summarized across cross-validation folds?

- RQ3: What do the results reveal about the current strengths and limitations of publicly available mpox skin-image datasets for reproducible AI benchmarking?

REVIEWER 2, POINT 2: Literature Gap and Specific Citations

------------------------------------------------------------

"The literature review doesn't provide comprehensive coverage. A few important pieces of literature about deep learning are not cited."

Specifically requested:

(a) DOI: 10.1007/s11042-024-18437-z (Monkeypox recognition using deep transfer learning)

(b) DOI: 10.1007/s00354-023-00227-0 (CNN-LSTM sentiment analysis on monkeypox tweets)

RESPONSE:

The literature gap is now explicitly articulated in the Introduction, supported by recent critiques from Hossain et al. (2025) and Vega et al. (2023). Both requested papers have been cited in the revised manuscript:

(a) Aslam et al. (2024, DOI: 10.1007/s11042-024-18437-z) is cited in the Related work section (Section 2) as a recent deep transfer-learning study on monkeypox visual recognition, further demonstrating the viability of pretrained networks in this domain.

(b) Khan et al. (2023, DOI: 10.1007/s00354-023-00227-0) is cited in the Introduction (Section 1) to illustrate the breadth of AI applications for mpox, which range from public-health surveillance through social-media sentiment analysis to diagnostic imaging from skin lesions.

Additionally, we have added Thieme et al. (2023, Nature Medicine) and Pramanik et al. (2023, PLOS ONE) to strengthen the literature coverage.

REVIEWER 2, POINT 3: Embedding Techniques

-------------------------------------------

"It is not clear how the authors used the embedding techniques. Please provide more explanations."

RESPONSE:

The revised manuscript uses standard transfer learning with seven pretrained backbones (ConvNeXt-Tiny, ViT-B/16, MobileNetV3-Large, ResNet-50, DenseNet-121, EfficientNet-B0, EfficientNetV2-S) rather than separate embedding-based approaches. Each backbone is initialized with ImageNet-1K V1 pretrained weights, and the final classification head is replaced with a binary output layer. The architecture of each backbone and the classification head modification are described in Section 3.4. The pretrained feature representations serve as the learned image embeddings, which are then adapted to the mpox classification task through end-to-end fine-tuning. No separate embedding extraction or retrieval-based classification is used in the main evaluation pipeline.

REVIEWER 2, POINT 4: Fine-Tuning Details

------------------------------------------

"It is not clear how the authors fine-tuned the model. Please add experiments and details."

RESPONSE:

Fine-tuning is now fully documented in Section 3.9:

- Main training: All backbone layers are unfrozen (no frozen layers). Per-backbone hyperparameters (optimizer, learning rate, weight decay, label smoothing, loss function, scheduler, warmup epochs, gradient clipping, drop-path rate) were selected by NSGA-II multi-objective optimization (840 trials, 6 epochs each) and are reported in Tables 2-3.

- Post-training fine-tuning: After main training, a short fine-tuning phase of 5 epochs at lr = 1e-5 was applied on original-only training data to address the augmented-to-original domain shift. All layers remained unfrozen. MixUp and CutMix augmentations were disabled during this phase. The scheduler was set to "none" to maintain a constant low learning rate.

- Early stopping: Patience = 4 epochs, monitored metric = f1_at_precision, applied on the validation set (not the test set).

- Both the pre-fine-tune checkpoint (best.pt) and post-fine-tune checkpoint (best_finetune.pt) are saved; the reported results use the post-fine-tune checkpoint.

REVIEWER 2, POINT 5: Parameter Comparison Unclear

---------------------------------------------------

"It is not clear how the parameters of the models are compared."

RESPONSE:

Table 4 now reports the number of trainable parameters and GFLOPs (floating-point operations per forward pass, computed using the torchprofile library) for all seven backbones at 224 x 224 input resolution with a binary classification head. This allows direct comparison of model complexity across architectures. For example, MobileNetV3-Large has the fewest parameters (3.0M) while ViT-B/16 has the most (85.8M). These complexity figures are discussed in the context of performance in the Discussion section.

REVIEWER 2, POINT 6: Tabular Comparison with 2023-2025 Works

--------------------------------------------------------------

"I would strongly advise including all major works in 2023 and 2024-25 and drawing a tabular comparison between your work and other works."

RESPONSE:

Table 1 now includes 12 representative peer-reviewed studies spanning 2022-2025:

- Sitaula and Shahi (2022), Sahin et al. (2022), Bala et al. (2023), Altun et al. (2023), Uysal (2023), Azar et al. (2023), Nayak et al. (2023, two studies), Almufareh et al. (2023), Munoz-Saavedra et al. (2023), Bamaqa et al. (2024), Elhadidy et al. (2025).

Each entry documents the task/dataset, split/validation strategy, best reported result, and the specific comparability limitation. The present study is included as the final row with a gray highlight. The narrative in Section 2 additionally discusses Thieme et al. (2023, Nature Medicine), Pramanik et al. (2023, PLOS ONE), Aslam et al. (2024), and Khan et al. (2023).

REVIEWER 2, POINT 7: Dataset Size Missing

-------------------------------------------

"There is no such sub-section mentioning the details about the dataset size."

RESPONSE:

Section 3.2 (Unified dataset construction) now fully documents:

- 876 original images (339 mpox, 537 non-mpox)

- 1,357 total images including augmented variants

- Class distribution: 38.7% mpox / 61.3% non-mpox

- Majority-class baseline accuracy: 0.613 (reported in Results)

- Sources: MSLD v1.0 and MSLD v2.0 with explicit class-mapping rules

- Grouping: 617 filename-derived groups

REVIEWER 2, POINT 8: Possible Improvements

--------------------------------------------

"I'd recommend adding some possible improvements for the proposed approach."

RESPONSE:

The Discussion section now identifies several specific improvement directions:

- Threshold optimization improved ensemble F1 by +0.008 compared to the default 0.5 threshold (F1 = 0.8255 vs. 0.8334), suggesting that adaptive threshold strategies merit further investigation.

- TTA provided a modest ensemble F1 gain of +0.009, indicating that more sophisticated test-time strategies (e.g., learned augmentation policies) could yield additional improvement.

- Fold-level variability (ensemble recall SD = 0.1119) suggests that performance is primarily constrained by data heterogeneity rather than architectural limitations, indicating that larger, more diverse datasets would be the most impactful improvement path.

- The mixed ECE results (temperature scaling improved calibration for ViT-B/16 and the ensemble but slightly worsened it for ConvNeXt-Tiny and MobileNetV3-Large) suggest that model-specific calibration strategies could improve overall calibration quality.

REVIEWER 2, POINT 9: Future Research Avenues

----------------------------------------------

"What are the avenues for future research or improvements identified based on the findings of this study?"

RESPONSE:

The Conclusion explicitly identifies four future research directions:

(1) External validation on independently collected clinical cohorts to establish generalizability beyond public datasets.

(2) Explicit patient-level deduplication using clinical metadata rather than filename-based grouping.

(3) Extension to richer multiclass differential diagnosis across multiple dermatological conditions.

(4) Prospective real-world assessment under realistic acquisition settings with diverse patient populations.

REVIEWER 2, POINT 10: Challenges in the Work

----------------------------------------------

"The challenges in the work need to be stated."

RESPONSE:

The Limitations paragraph in the Discussion now enumerates five specific challenges:

(1) The benchmark relies on public lesion images rather than prospectively collected clinical data; external validation remains essential before deployment. This is mitigated by a five-point internal robustness suite (pHash audit, ECE analysis, threshold verification, TTA ablation, statistical testing).

(2) Perceptual-hash auditing cannot detect semantically similar images that differ at the pixel level (e.g., the same lesion from slightly different angles); true patient-level identity control would require metadata not available in MSLD.

(3) The benchmark is binary (mpox vs. non-mpox), whereas real clinical workflows require multiclass differential diagnosis.

(4) The absence of image-level skin-tone annotations prevents direct fairness assessment across skin tones.

(5) All robustness analyses are internal to the same public data; their generalizability to unseen clinical populations remains to be established.

REVIEWER 2, POINT 11: Challenges in the Dataset

-------------------------------------------------

"What are the challenges in the dataset?"

RESPONSE:

Section 3.3 and the Discussion now document three specific dataset challenges:

(a) The perceptual-hash audit revealed that MSLD v1.0 and v2.0 share a substantial overlapping core: 95 near-duplicate pairs were identified, confirming that naive merging of the two dataset versions without content-level deduplication would inflate apparent performance.

(b) Filename-based grouping identified 617 groups with up to 14 images per group, indicating that some lesions are represented by multiple images that must be kept in the same cross-validation fold to prevent leakage.

(c) The Discussion notes that fold-level variability is primarily attributable to source-level heterogeneity (different acquisition devices, lighting conditions, and racial diversity profiles between MSLD v1.0 and v2.0) and to the small per-fold test sizes (approximately 68 positive and 107 negative images per fold).
