# Supplementary material for: AI-driven diagnosis of mpox using deep learning models
Source: PLoS One. 2026 Jul 10;21(7):e0352161. doi: 10.1371/journal.pone.0352161 (PMC13354086; doi:10.1371/journal.pone.0352161)
Supplement: Attachment — Submitted filename: Response_to_Editor_R2.docx [file pone.0352161.s002.docx]

RESPONSE TO THE ACADEMIC EDITOR

Manuscript ID: PONE-D-25-66974_R1

Title: AI-Driven Diagnosis of Mpox using Deep Learning Models

Authors: Aboshosha et al.

Academic Editor: Dr. Morufu Olalekan Raimi

Decision: Minor Revision

Dear Dr. Raimi,

We thank you for the careful and constructive evaluation of our revised manuscript and for the positive assessment of the methodological restructuring. We are grateful for the recommendation toward acceptance. We have addressed each of the required minor revisions point by point below. For every item we indicate the action taken and the location in the revised manuscript. The editor's comments are reproduced first, followed by our response.

Throughout this letter, all table and figure references use the final manuscript numbering, which we have re-verified against the compiled document. For convenience, the final table numbering is summarized at the end of this letter.

Sincerely,

Ibrahim Sadek, on behalf of all co-authors

Corresponding author

**RESPONSE TO JOURNAL REQUIREMENTS**

Reference list completeness and retraction check. We have reviewed the full reference list for completeness and accuracy. Every in-text citation resolves to a complete bibliographic entry (author, title, venue, year, and DOI where applicable), and there are no orphaned, duplicated, or missing references. We additionally checked the cited DOIs against Crossref and the Retraction Watch database; to the best of our knowledge none of the cited works has been retracted or carries an expression of concern. Consequently, no references were removed or replaced on these grounds, and no retraction notices are required. Should any item be identified as retracted during production, we will indicate its retracted status in the reference list and add a full citation to the corresponding retraction notice, as required.

**REQUIRED MINOR REVISIONS**

**COMMENT 1 - Title inconsistency**

Editor: The main manuscript uses "Mpox" and "Monkeypox" interchangeably in the title and abstract. Please standardize to "Mpox" throughout, consistent with current WHO terminology.

Response: We have standardized the disease terminology to "Mpox" in line with current WHO nomenclature.

- The title now reads "AI-Driven Diagnosis of Mpox using Deep Learning Models."

- The abstract and body text now use "mpox" consistently, including the previously inconsistent descriptive passages in the Related Work section (e.g., "mpox detection from skin lesion images" and "mpox recognition from visual images") and the class listing in the comparison table.

For accuracy, we have retained "monkeypox" only where it is scientifically correct or verbatim, namely: (i) the formal virus name "monkeypox virus (MPXV)," which WHO continues to use for the pathogen; (ii) literal dataset folder labels reproduced from the public MSLD repositories (e.g., "Monkey Pox", "Monkeypox"), which must remain verbatim for reproducibility; (iii) the proper model name "MonkeyNet"; and (iv) bibliographic citation keys. We trust this distinction is consistent with the editor's intent.

**COMMENT 2 - Table numbering discrepancies**

Editor: In the point-by-point response, the authors refer to "Table 3" for augmentation parameters and "Table 4" for complexity, but in the manuscript, augmentation hyperparameters appear in Table 5 and complexity in Table 7. Please reconcile all cross-references in the response letter to match the final manuscript's table numbering.

Response: We apologize for the inconsistency in the previous response letter. We have re-verified the table numbering in the final compiled manuscript and corrected all cross-references accordingly. In the final manuscript:

- Augmentation and regularization hyperparameters appear in Table 5 ("Selected backbone-specific augmentation and regularization hyperparameters").

- Model complexity appears in Table 7 ("Model complexity of the evaluated backbones").

All table references in this response letter now match the final manuscript numbering (see the summary at the end of this letter). No further table-numbering inconsistencies remain.

**COMMENT 3 - Figure 1 caption clarity**

Editor: Figure 1 (dot and whisker plot) is well constructed, but the caption should explicitly state that points are mean values across five folds and whiskers represent +/- 1 standard deviation.

Response: We have revised the caption of Figure 1 to state this explicitly. It now reads:

"Cross-validation performance summary of the evaluated methods across five folds. Each point represents the mean value across the five cross-validation folds, and the whiskers represent +/- 1 standard deviation around that mean. Methods are ordered by mean F1-score."

**COMMENT 4 - External validation statement**

Editor: The Discussion correctly lists the lack of external validation as a limitation. However, the Conclusion currently states that "future work should extend this line of research through externally validated datasets." Please add a one-sentence acknowledgment in the Abstract as well, e.g., "External clinical validation remains necessary before deployment."

Response: We have added the requested acknowledgment to the end of the Abstract, phrased as a logically linked closing statement rather than a standalone sentence so that it follows naturally from the methodological framing. The Abstract now closes with:

"...while highlighting the limitations of current public skin-image data. Accordingly, these results should be interpreted as a reproducible reference benchmark rather than a clinically validated diagnostic tool, and external clinical validation remains necessary before deployment."

This complements the existing statements in the Discussion and Conclusion.

**COMMENT 5 - Minor typographical corrections**

Editor (a): "originally test evaluation" -> "original only test evaluation".

Response: The phrasing throughout the manuscript now reads "original-only test evaluation" (e.g., in the Abstract, Related Work, Methods, Results, and Discussion). The flagged typographic form is no longer present.

Editor (b): Ensure consistent wording ("16 fold" vs. "x16") for test-time augmentation across the manuscript.

Response: We have standardized the wording to "16-fold" throughout. Specifically:

- The shared training-and-evaluation settings table now lists the test-time augmentation value as "16-fold."

- The TTA ablation table caption now reads "Effect of 16-fold test-time augmentation (TTA) on mean F1-score and AUC across five folds," and its column header reads "TTA on (16-fold)."

- The running text already used "16-fold," so the terminology is now uniform.

**SUMMARY OF FINAL MANUSCRIPT TABLE NUMBERING (for reference)**

Table 1 - Standardized comparison of representative peer-reviewed mpox skin-image studies

Table 2 - Unified dataset construction rules

Table 3 - Shared training and evaluation settings (includes the TTA = 16-fold row)

Table 4 - Selected backbone-specific optimization and loss hyperparameters

Table 5 - Selected backbone-specific AUGMENTATION and regularization hyperparameters

Table 6 - Fold-wise test performance (mean +/- SD)

Table 7 - Model COMPLEXITY of the evaluated backbones

Table 8 - Mean confusion-matrix components

Table 9 - Normalized ensemble weights per fold

Table 10 - Expected Calibration Error before/after temperature scaling

Table 11 - Validation-selected decision thresholds and test precision/recall

Table 12 - Effect of 16-fold test-time augmentation (TTA) on F1 and AUC

Figure 1 is the dot-and-whisker cross-validation performance summary.

We believe these revisions fully address the remaining points raised. We thank you again for the constructive review, which has further improved the clarity and consistency of the manuscript.
