# Supplementary material for: AI-driven diagnosis of mpox using deep learning models
Source: PLoS One. 2026 Jul 10;21(7):e0352161. doi: 10.1371/journal.pone.0352161 (PMC13354086; doi:10.1371/journal.pone.0352161)
Supplement: Peer review history

# Peer review: Decision Letter 0

**DOI:** 10.1371/journal.pone.0352161.r001

**Authors:** Morufu Olalekan Raimi

11 Mar 2026

PONE-D-25-66974

AI-Driven Diagnosis of Monkeypox using Deep Learning Models

PLOS One

Dear Dr. Sadek,

Thank you for submitting your manuscript to PLOS ONE. After careful consideration, we feel that it has merit but does not fully meet PLOS ONE’s publication criteria as it currently stands. Therefore, we invite you to submit a revised version of the manuscript that addresses the points raised during the review process.

Please submit your revised manuscript by Apr 25 2026 11:59PM. If you will need more time than this to complete your revisions, please reply to this message or contact the journal office at plosone@plos.org. When you're ready to submit your revision, log on to https://www.editorialmanager.com/pone/ and select the 'Submissions Needing Revision' folder to locate your manuscript file.

Please include the following items when submitting your revised manuscript:

- A letter that responds to each point raised by the academic editor and reviewer(s). You should upload this letter as a separate file labeled 'Response to Reviewers'.
- A marked-up copy of your manuscript that highlights changes made to the original version. You should upload this as a separate file labeled 'Revised Manuscript with Track Changes'.
- An unmarked version of your revised paper without tracked changes. You should upload this as a separate file labeled 'Manuscript'.

If you would like to make changes to your financial disclosure, please include your updated statement in your cover letter. Guidelines for resubmitting your figure files are available below the reviewer comments at the end of this letter.

If applicable, we recommend that you deposit your laboratory protocols in protocols.io to enhance the reproducibility of your results. Protocols.io assigns your protocol its own identifier (DOI) so that it can be cited independently in the future. For instructions see: https://journals.plos.org/plosone/s/submission-guidelines#loc-laboratory-protocols. Additionally, PLOS ONE offers an option for publishing peer-reviewed Lab Protocol articles, which describe protocols hosted on protocols.io. Read more information on sharing protocols at https://plos.org/protocols?utm_medium=editorial-email&utm_source=authorletters&utm_campaign=protocols.

We look forward to receiving your revised manuscript.

Kind regards,

Morufu Olalekan Raimi, Ph.D

Academic Editor

PLOS One

Journal Requirements:

When submitting your revision, we need you to address these additional requirements.

1. Please ensure that your manuscript meets PLOS ONE's style requirements, including those for file naming. The PLOS ONE style templates can be found at

https://journals.plos.org/plosone/s/file?id=wjVg/PLOSOne_formatting_sample_main_body.pdf and https://journals.plos.org/plosone/s/file?id=ba62/PLOSOne_formatting_sample_title_authors_affiliations.pdf

2. Please note that PLOS One has specific guidelines on code sharing for submissions in which author-generated code underpins the findings in the manuscript. In these cases, we expect all author-generated code to be made available without restrictions upon publication of the work. Please review our guidelines at https://journals.plos.org/plosone/s/materials-and-software-sharing#loc-sharing-code and ensure that your code is shared in a way that follows best practice and facilitates reproducibility and reuse.

3. We note that your Data Availability Statement is currently as follows: [All relevant data are within the manuscript and its Supporting Information files]

Please confirm at this time whether or not your submission contains all raw data required to replicate the results of your study. Authors must share the “minimal data set” for their submission. PLOS defines the minimal data set to consist of the data required to replicate all study findings reported in the article, as well as related metadata and methods (https://journals.plos.org/plosone/s/data-availability#loc-minimal-data-set-definition).

For example, authors should submit the following data:

- The values behind the means, standard deviations and other measures reported;

- The values used to build graphs;

- The points extracted from images for analysis.

Authors do not need to submit their entire data set if only a portion of the data was used in the reported study.

If your submission does not contain these data, please either upload them as Supporting Information files or deposit them to a stable, public repository and provide us with the relevant URLs, DOIs, or accession numbers. For a list of recommended repositories, please see https://journals.plos.org/plosone/s/recommended-repositories.

If there are ethical or legal restrictions on sharing a de-identified data set, please explain them in detail (e.g., data contain potentially sensitive information, data are owned by a third-party organization, etc.) and who has imposed them (e.g., an ethics committee). Please also provide contact information for a data access committee, ethics committee, or other institutional body to which data requests may be sent. If data are owned by a third party, please indicate how others may request data access.

4. We note that Figure 1 in your submission contains map images which may be copyrighted. All PLOS content is published under the Creative Commons Attribution License (CC BY 4.0), which means that the manuscript, images, and Supporting Information files will be freely available online, and any third party is permitted to access, download, copy, distribute, and use these materials in any way, even commercially, with proper attribution. For these reasons, we cannot publish previously copyrighted maps or satellite images created using proprietary data, such as Google software (Google Maps, Street View, and Earth). For more information, see our copyright guidelines: http://journals.plos.org/plosone/s/licenses-and-copyright.

We require you to either (1) present written permission from the copyright holder to publish these figures specifically under the CC BY 4.0 license, or (2) remove the figures from your submission:

1. You may seek permission from the original copyright holder of Figure 1 to publish the content specifically under the CC BY 4.0 license.

We recommend that you contact the original copyright holder with the Content Permission Form (http://journals.plos.org/plosone/s/file?id=7c09/content-permission-form.pdf) and the following text:

“I request permission for the open-access journal PLOS ONE to publish XXX under the Creative Commons Attribution License (CCAL) CC BY 4.0 (http://creativecommons.org/licenses/by/4.0/). Please be aware that this license allows unrestricted use and distribution, even commercially, by third parties. Please reply and provide explicit written permission to publish XXX under a CC BY license and complete the attached form.”

Please upload the completed Content Permission Form or other proof of granted permissions as an "Other" file with your submission.

In the figure caption of the copyrighted figure, please include the following text: “Reprinted from [ref] under a CC BY license, with permission from [name of publisher], original copyright [original copyright year].”

2. If you are unable to obtain permission from the original copyright holder to publish these figures under the CC BY 4.0 license or if the copyright holder’s requirements are incompatible with the CC BY 4.0 license, please either i) remove the figure or ii) supply a replacement figure that complies with the CC BY 4.0 license. Please check copyright information on all replacement figures and update the figure caption with source information. If applicable, please specify in the figure caption text when a figure is similar but not identical to the original image and is therefore for illustrative purposes only.

The following resources for replacing copyrighted map figures may be helpful:

USGS National Map Viewer (public domain): http://viewer.nationalmap.gov/viewer/

The Gateway to Astronaut Photography of Earth (public domain): http://eol.jsc.nasa.gov/sseop/clickmap/

Maps at the CIA (public domain): https://www.cia.gov/library/publications/the-world-factbook/index.html and https://www.cia.gov/library/publications/cia-maps-publications/index.html

NASA Earth Observatory (public domain): http://earthobservatory.nasa.gov/

Landsat: http://landsat.visibleearth.nasa.gov/

USGS EROS (Earth Resources Observatory and Science (EROS) Center) (public domain): http://eros.usgs.gov/#

Natural Earth (public domain): http://www.naturalearthdata.com/

5. We note that Figures 1 (Figure 1 Monkeypox clinic's website), 2 (Figure 2 Sample images from the study dataset), 3, 4, 5, and 7 in your submission contain copyrighted images. All PLOS content is published under the Creative Commons Attribution License (CC BY 4.0), which means that the manuscript, images, and Supporting Information files will be freely available online, and any third party is permitted to access, download, copy, distribute, and use these materials in any way, even commercially, with proper attribution. For more information, see our copyright guidelines: http://journals.plos.org/plosone/s/licenses-and-copyright.

We require you to either (1) present written permission from the copyright holder to publish these figures specifically under the CC BY 4.0 license, or (2) remove the figures from your submission:

1. You may seek permission from the original copyright holder of Figures 1 (Figure 1 Monkeypox clinic's website), 2 (Figure 2 Sample images from the study dataset), 3, 4, 5, and 7 to publish the content specifically under the CC BY 4.0 license.

We recommend that you contact the original copyright holder with the Content Permission Form (http://journals.plos.org/plosone/s/file?id=7c09/content-permission-form.pdf) and the following text:

“I request permission for the open-access journal PLOS ONE to publish XXX under the Creative Commons Attribution License (CCAL) CC BY 4.0 (http://creativecommons.org/licenses/by/4.0/). Please be aware that this license allows unrestricted use and distribution, even commercially, by third parties. Please reply and provide explicit written permission to publish XXX under a CC BY license and complete the attached form.”

Please upload the completed Content Permission Form or other proof of granted permissions as an "Other" file with your submission.

In the figure caption of the copyrighted figure, please include the following text: “Reprinted from [ref] under a CC BY license, with permission from [name of publisher], original copyright [original copyright year].”

2. If you are unable to obtain permission from the original copyright holder to publish these figures under the CC BY 4.0 license or if the copyright holder’s requirements are incompatible with the CC BY 4.0 license, please either i) remove the figure or ii) supply a replacement figure that complies with the CC BY 4.0 license. Please check copyright information on all replacement figures and update the figure caption with source information. If applicable, please specify in the figure caption text when a figure is similar but not identical to the original image and is therefore for illustrative purposes only.

6. Please renumber your figures and update the corresponding citations in the text to ensure they follow a sequential order.

If the reviewer comments include a recommendation to cite specific previously published works, please review and evaluate these publications to determine whether they are relevant and should be cited. There is no requirement to cite these works unless the editor has indicated otherwise.

Additional Editor Comments:

PLOS ONE Editorial Decision

Manuscript Number: PONE-D-25-66974

Title: AI-Driven Diagnosis of Monkeypox using Deep Learning Models

Authors: Bassam W. Aboshosha, Shafiq Ul Rehman, Lamees N. Mahmoud, Ibrahim Sadek

Dear Dr. Sadek and colleagues,

Thank you for submitting your manuscript to PLOS ONE. I have now completed a thorough assessment of your submission, including the two reviewer reports and my own detailed evaluation. The manuscript addresses a timely and clinically relevant topic, the application of deep learning for mpox diagnosis using skin image analysis. The proposed integration of this diagnostic capability into an “unmanned smart clinic” concept demonstrates awareness of real-world deployment challenges, particularly in resource-limited settings. These elements represent genuine strengths. However, after careful review, I must concur with the reviewers that the manuscript in its current form does not meet the standards for publication in PLOS ONE. The concerns raised are fundamental and extend beyond minor revision.

Summary of Critical Issues

1. The Methodological Contribution is Insufficient

Reviewer 1's assessment is direct and, in my judgment, accurate: “There is no novelty in the methodology. This study is totally relied on established architectures without any significant advancement.” The manuscript evaluates five standard pre-trained CNN architectures (MobileNet, DenseNet121, ResNet50, Inception-v3, EfficientNet) using a publicly available Kaggle dataset. The core methodology, transfer learning with fine-tuning on medical images, is well-established and has been extensively applied to mpox detection in the literature, as the authors' own extensive reference list demonstrates (over 45 cited studies on this exact topic).

The critical question: What does this study add that is not already present in the existing literature? The authors do not articulate a clear methodological innovation, a novel architectural modification, a new training strategy, or a substantive advance in how these models are applied. Without such a contribution, the manuscript reads as a replication study, which, while potentially valuable, does not constitute the original research expected by PLOS ONE.

2. The Literature Review is Incomplete and the Contribution is Unclear

Reviewer 2 correctly notes that the literature review "doesn't provide comprehensive coverage of related work" and identifies a “significant gap in coverage of the state-of-the-art.”

This criticism is particularly salient given the sheer volume of existing work. The authors cite over 45 studies on AI-based mpox detection, yet the manuscript does not:

• Synthesize what these studies have collectively established

• Identify persistent gaps or limitations in the existing literature

• Clearly articulate how the present study addresses those gaps

• Demonstrate why another comparative evaluation of standard models is needed

The introduction states the study's contributions as: (1) developing AI-driven diagnostic models, (2) proposing a smart unmanned medical clinic concept, (3) presenting a comparative analysis, and (4) supporting AI-enhanced healthcare. However:

• Contribution (1) is not novel, dozens of studies have already developed such models

• Contribution (2) is a conceptual proposal, not empirically validated in this study

• Contribution (3) is descriptive, not analytical, it does not generate new insights about why certain models perform better or how they could be improved

• Contribution (4) is a general aspiration, not a specific finding

The manuscript needs a clearly articulated research gap and a specific research question that this study answers. Without this, the contribution remains unclear.

3. The Dataset Description is Inadequate

Reviewer 2 notes: “There is no such sub-section mentioning the details about the dataset size. I couldn't find anything regarding the size or quantity of the dataset.”

This is a serious omission. Table 2 provides class distributions for training, validation, and test sets, but critical information is missing:

• Source: The authors state the dataset was obtained from Kaggle and “has previously been used in research.” Which specific Kaggle dataset? What is its original source? How were images collected? What are the inclusion/exclusion criteria?

• Verification: “Each image of a skin lesion was double-checked using references and Google's reverse image search.” This is insufficient for clinical-grade data. What were the verification criteria? Who performed the verification? What was the inter-rater reliability?

• Characteristics: What are the image resolutions? What are the demographic characteristics of the patients represented? What are the lesion locations, stages, and presentations? Are there multiple images per patient?

• Confounders: Are there potential confounders such as image quality differences between classes, background variations, or lighting conditions that could introduce bias?

Without this information, the validity of the results cannot be assessed. The near-perfect performance of EfficientNet (99.64% accuracy) raises concerns about potential data leakage, overfitting, or dataset biases that cannot be evaluated without detailed dataset documentation.

4. The Experimental Details are Insufficient for Reproducibility

Reviewer 2 raises multiple concerns about experimental transparency:

• “It is not clear how the authors used the embedding techniques.”

• “It is not clear how the authors fine-tuned the model.”

• “It is not clear how the parameters of the models are compared.”

Critical missing information includes:

• Preprocessing details: Beyond “poor-quality images were eliminated,” what were the specific criteria? What preprocessing steps were applied (resizing, normalization, color space conversion)?

• Augmentation details: Which specific augmentations were applied? With what parameters? Was augmentation applied only to training data, or also to validation/test data?

• Training details: What was the optimization algorithm? Learning rate? Batch size? Number of epochs? Early stopping criteria? Loss function?

• Fine-tuning details: Which layers were frozen vs. fine-tuned? What were the initial and final layer configurations?

• Hardware/software: What computing infrastructure was used? What frameworks and versions?

• Hyperparameter selection: How were hyperparameters chosen? Was there a validation-based selection process?

Without these details, the experiments cannot be reproduced or critically evaluated.

5. The Reported Performance Raises Concerns

EfficientNet achieving 99.64% validation accuracy with 100% precision and recall (zero false positives, zero false negatives) on a medical image classification task is extraordinarily high.

This level of performance warrants critical scrutiny:

• Class imbalance: The dataset is nearly balanced (1,428 mpox, 1,376 normal), so imbalance is not an explanation.

• Dataset size: 2,804 images is modest for deep learning. With perfect performance, one must consider whether the test set is too easy, whether there is data leakage between train/validation/test, or whether the dataset contains systematic biases that the model has learned.

• Comparison to literature: The authors' own Table 4 shows that most published studies report accuracies in the 82-98% range, with only a few achieving >99%. What explains this study's superior performance?

The manuscript does not address these concerns. There is no analysis of difficult cases, no error analysis (because there are no errors), no discussion of potential dataset limitations, and no external validation.

6. The “Unmanned Smart Clinic” Concept is Underdeveloped

The proposal of an unmanned smart clinic is conceptually interesting but:

• It is not integrated with the core analysis. The clinic concept is described in Section 3, but the AI models are evaluated in isolation. There is no demonstration or validation of how these models would function within such a clinic.

• Operational details are missing. How would image capture be standardized? What would prevent users from submitting poor-quality images? How would the system handle uncertainty or edge cases? What would be the workflow for positive vs. negative results?

• Implementation challenges are not addressed. Connectivity, data privacy, regulatory approval, user acceptance, and integration with existing health systems are not discussed.

As presented, this section reads as a conceptual illustration rather than a substantive contribution.

7. The Literature Comparison Table is Problematic

Table 4 attempts to compare this study with existing work, but it contains multiple issues:

• Inconsistent reporting: Some entries report accuracy with percentages, others without. Some include confidence intervals, most do not.

• Missing context: The table does not indicate dataset sizes, class distributions, or validation methodologies, making comparisons meaningless.

• Self-citation: The table includes “This study” at the end, but the comparison is superficial.

• Errors: Reference numbering in the table appears inconsistent with the reference list.

A proper comparison would require standardized metrics, dataset characteristics, and validation approaches, none of which are provided.

8. The Writing and Presentation Require Substantial Improvement

Beyond the methodological issues, the manuscript has significant presentation problems:

• Redundancy: The introduction repeats information about mpox symptoms and transmission across multiple paragraphs.

• Organization: Section 4 (“Methodology”) includes subsections that could be better structured. The mathematical notation in lines 46-136 appears to be from a different manuscript (it references “this section” and includes theorems that are not used in the analysis).

• Figures: Figures 8-14 are difficult to interpret. Some are low-resolution, others have illegible text. The precision-recall curves lack threshold annotations.

• Table formatting: Table 1 extends across multiple pages with inconsistent formatting. Table 3 presents metrics without confidence intervals.

• References: The reference list is overly long (48 references) but not well-integrated into the text. Many references are cited in blocks without specific attribution.

Specific Required Revisions

If the authors wish to resubmit, the following must be addressed comprehensively. This represents a major revision at minimum, and the authors should carefully consider whether the current study can be revised to meet the required standards.

A. Clarify the Novelty and Contribution (Required)

• Conduct a thorough literature review and clearly articulate the specific gap this study addresses.

• State explicit research questions or hypotheses.

• Demonstrate what this study adds beyond existing work, methodological innovation, new insights, or practical advances.

B. Provide Complete Dataset Documentation (Required)

• Specify the exact Kaggle dataset (URL, version, source).

• Provide detailed inclusion/exclusion criteria for image selection.

• Report image characteristics (resolution, format, quality metrics).

• Describe the verification process in detail, including who performed it and how reliability was ensured.

• Disclose any potential confounders or biases in the dataset.

C. Provide Complete Experimental Details (Required)

• Document all preprocessing steps with specific parameters.

• List all augmentation techniques with parameters and rationale.

• Report training hyperparameters (optimizer, learning rate, batch size, epochs, loss function).

• Describe fine-tuning strategy (which layers frozen, learning rates for different layers).

• Specify hardware and software environment.

• Detail the hyperparameter selection process.

D. Address Performance Concerns (Required)

• Conduct and report cross-validation results (not just a single train/validation/test split).

• Provide confidence intervals for all performance metrics.

• Report performance on multiple metrics, including sensitivity, specificity, PPV, NPV, and F1-score for each class.

• Analyze difficult cases, what images were misclassified (if any) and why?

• If performance remains perfect, discuss potential explanations and limitations.

E. Validate on External Data (Strongly Recommended)

• Test the models on an independent dataset not used in development.

• If external validation is not possible, explicitly discuss this as a major limitation.

F. Develop the Clinic Concept or Remove It (Required)

• Either integrate the clinic concept with empirical validation (e.g., testing the models under simulated clinic conditions, analyzing image quality variations), or remove it and focus on the core diagnostic analysis.

• If retained, address operational, regulatory, and implementation challenges.

G. Revise the Literature Comparison (Required)

• Create a meaningful comparison table with standardized metrics and dataset characteristics.

• Discuss why performance differs across studies.

• Acknowledge limitations in cross-study comparisons.

H. Improve Writing and Presentation (Required)

• Restructure the manuscript with clear sections: Introduction, Related Work, Methods, Results, Discussion, Conclusion.

• Remove redundant content.

• Ensure all figures are high-resolution with legible text and self-explanatory captions.

• Reformatted tables for clarity and consistency.

• Review and revise references to ensure accurate citation and formatting per PLOS ONE style.

I. Address Reviewer 2's Specific Concerns (Required)

• Add objectives and motivations tied to literature gaps.

• Add research questions.

• Cite and discuss missing relevant literature (e.g., the works suggested by Reviewer 2).

• Discuss challenges in the work and the dataset.

• Identify avenues for future research.

Decision: Major Revision Required

Given the above, I cannot recommend acceptance in the current form. The manuscript does not meet PLOS ONE's standards for methodological rigor, novelty, transparency, or reproducibility.

The authors may choose to resubmit a substantially revised manuscript addressing all of the concerns outlined above. Any resubmission will be evaluated de novo and will be sent for additional peer review. Given the fundamental nature of the concerns, particularly regarding novelty, the authors should carefully consider whether the revised manuscript can demonstrate a clear contribution. If the authors believe these concerns cannot be adequately addressed, they may wish to consider alternative venues more appropriate for replication studies or methodological validations. I thank the authors for their work on an important public health problem and encourage them to consider how their approach might be advanced to make a more distinctive contribution.

Sincerely,

Morufu Olalekan Raimi, PhD

Academic Editor

PLOS ONE

[Note: HTML markup is below. Please do not edit.]

Reviewers' comments:

Reviewer's Responses to Questions

**Comments to the Author**

1. Is the manuscript technically sound, and do the data support the conclusions?

The manuscript must describe a technically sound piece of scientific research with data that supports the conclusions. Experiments must have been conducted rigorously, with appropriate controls, replication, and sample sizes. The conclusions must be drawn appropriately based on the data presented.

Reviewer #1: No

Reviewer #2: Yes

**********

2. Has the statistical analysis been performed appropriately and rigorously?

Reviewer #1: No

Reviewer #2: N/A

**********

3. Have the authors made all data underlying the findings in their manuscript fully available?

The PLOS Data policy requires authors to make all data underlying the findings described in their manuscript fully available without restriction, with rare exception (please refer to the Data Availability Statement in the manuscript PDF file). The data should be provided as part of the manuscript or its supporting information, or deposited to a public repository. For example, in addition to summary statistics, the data points behind means, medians and variance measures should be available. If there are restrictions on publicly sharing data—e.g. participant privacy or use of data from a third party—those must be specified.

Reviewer #1: No

Reviewer #2: Yes

**********

4. Is the manuscript presented in an intelligible fashion and written in standard English?

PLOS ONE does not copyedit accepted manuscripts, so the language in submitted articles must be clear, correct, and unambiguous. Any typographical or grammatical errors should be corrected at revision, so please note any specific errors here.

Reviewer #1: No

Reviewer #2: Yes

**********

5. Review Comments to the Author

Please use the space provided to explain your answers to the questions above. You may also include additional comments for the author, including concerns about dual publication, research ethics, or publication ethics. (Please upload your review as an attachment if it exceeds 20,000 characters)

Reviewer #1: There is no novelty in the methodology. This study is totally relied on established architectures without any significant advancement. The paper is not suitable for a reputable journal like plos one.

Reviewer #2: The manuscript presents several significant issues that need to be addressed before it can be considered for publication. Please find my detailed comments and concerns below:

1. Please add two paragraphs in the introduction: a) objectives and motivations tied to gaps in the literature; b) research questions.

2. The literature review of the article doesn't provide comprehensive coverage of related work. Most troublesome is the significant gap in coverage of the state-of-the-art. A few important pieces of literature about deep learning are not cited, for example.

(a). Monkeypox recognition and prediction from visuals using deep transfer learning-based neural networks, DOI: https://link.springer.com/article/10.1007/s11042-024-18437-z

3. It is not clear how the authors used the embedding techniques. Please provide more explanations.

4. It is not clear how the authors fine-tuned the model. Please add experiments and details.

5. It is not clear how the parameters of the models are compared.

6. I would strongly advise including all major works in 2023 and 2024-25 and drawing a tabular comparison between your work and other works. A few works worth comparing, referring to, or citing are below:

(b). A CNN-LSTM-Based Hybrid Deep Learning Approach for Sentiment Analysis on Monkeypox Tweets

DOI: https://link.springer.com/article/10.1007/s00354-023-00227-0

7. There is no such sub-section mentioning the details about the dataset size. I couldn’t find anything regarding the size or quantity of the dataset.

8. I'd recommend adding some possible improvements for the proposed approach.

9. What are the avenues for future research or improvements identified based on the findings of this study?

10. The challenges in the work need to be stated (As mentioned in the title).

11. What are the challenges in the dataset?

**********

6. PLOS authors have the option to publish the peer review history of their article (what does this mean?). If published, this will include your full peer review and any attached files.

If you choose “no”, your identity will remain anonymous but your review may still be made public.

**Do you want your identity to be public for this peer review?**  For information about this choice, including consent withdrawal, please see our Privacy Policy.

Reviewer #1: **Yes:**  Dr. Tayyaba Anees

Reviewer #2: **Yes:**  Dr. Gaurav Meena

**********

[NOTE: If reviewer comments were submitted as an attachment file, they will be attached to this email and accessible via the submission site. Please log into your account, locate the manuscript record, and check for the action link "View Attachments". If this link does not appear, there are no attachment files.]

To ensure your figures meet our technical requirements, please review our figure guidelines: https://journals.plos.org/plosone/s/figures

You may also use PLOS’s free figure tool, NAAS, to help you prepare publication quality figures: https://journals.plos.org/plosone/s/figures#loc-tools-for-figure-preparation.

NAAS will assess whether your figures meet our technical requirements by comparing each figure against our figure specifications.

# Author comment: Author response to Decision Letter 1

**DOI:** 10.1371/journal.pone.0352161.r002

27 Apr 2026

POINT-BY-POINT RESPONSE TO EDITOR AND REVIEWER COMMENTS

=========================================================

Manuscript ID: PONE-D-25-66974

Manuscript Title: AI-Driven Diagnosis of Monkeypox using Deep Learning Models

We sincerely thank the Associate Editor, Dr. Morufu Olalekan Raimi, and the two Reviewers for their thorough and constructive evaluation. Every concern has been carefully considered and has led to a substantially revised manuscript. Below we provide a detailed point-by-point response. All referenced sections, tables, and figures refer to the revised manuscript.

=========================================================

RESPONSE TO ASSOCIATE EDITOR

=========================================================

EDITOR CONCERN 1: Insufficient Methodological Contribution

-----------------------------------------------------------

"There is no novelty in the methodology. This study is totally relied on established architectures without any significant advancement."

RESPONSE:

We fully agree that the former manuscript did not clearly articulate its contribution and read as a replication study. The revised manuscript has been completely restructured. The contribution is now explicitly framed as methodological rather than architectural. The study does not propose a new backbone; instead, it provides a unified curation recipe, a leakage-aware validation design, and fold-wise reference results intended to support defensible comparison with prior and future mpox skin-image studies.

Specifically, the revised Introduction (Section 1) states three objectives: (1) construct a unified curated binary dataset with fully specified class-mapping and selection rules; (2) compare multiple pretrained models and a weighted ensemble under a shared pipeline; and (3) examine how dataset construction and leakage-aware evaluation influence the interpretation of performance on public mpox lesion data. Three research questions (RQ1-RQ3) are enumerated to precisely define the scope.

This repositioning addresses the novelty concern by delineating what the study contributes (a rigorous, auditable benchmark design) from what it does not claim (a new architecture).

EDITOR CONCERN 2: Literature Review Incomplete, Contribution Unclear

---------------------------------------------------------------------

"The literature review doesn't provide comprehensive coverage of related work. Most troublesome is the significant gap in coverage of the state-of-the-art."

RESPONSE:

Section 2 (Related work) has been entirely rewritten. It now includes a narrative review of 14 peer-reviewed studies spanning 2022-2025, covering transfer learning, explainable AI, ensemble methods, mobile deployment, and optimization-based approaches. A new standardized comparison table (Table 1) presents 12 representative studies with five columns: Study, Task/dataset, Split/validation, Best reported result, and Comparability limitation. Each row explicitly explains why direct comparison with the present benchmark is limited, preventing misleading leaderboard-style comparisons.

The research gap is now clearly identified in the Introduction: heterogeneous benchmarking practices prevent meaningful comparison across studies. This is supported by recent critiques from Hossain et al. (2025), who highlighted dataset quality and inconsistent benchmarking as major barriers, and Vega et al. (2023), who showed that some early mpox datasets contained flawed or medically irrelevant web-scraped content.

The following studies have been added to strengthen the coverage:

- Thieme et al. (2023, Nature Medicine): deep-learning classification of mpox skin lesions with geographically diverse clinical validation

- Pramanik et al. (2023, PLOS ONE): CNN amalgamation with Beta function-based normalization

- Aslam et al. (2024): deep transfer-learning approach for monkeypox visual recognition (as requested by Reviewer 2)

- Khan et al. (2023): CNN-LSTM sentiment analysis on monkeypox tweets (as requested by Reviewer 2)

- Elhadidy et al. (2025): multiclass benchmarking on MSLD v2.0

EDITOR CONCERN 3: Dataset Description Inadequate

-------------------------------------------------

"There is no such sub-section mentioning the details about the dataset size. I couldn't find anything regarding the size or quantity of the dataset."

RESPONSE:

Section 3.2 (Unified dataset construction) now provides complete documentation:

- Sources: MSLD v1.0 (cited via Almufareh et al. 2023) and MSLD v2.0 (cited via Ali et al. 2024), both publicly available repositories.

- Size: 876 original images (339 mpox, 537 non-mpox) and 1,357 total images including augmented variants.

- Class distribution: 38.7% mpox / 61.3% non-mpox. The majority-class baseline accuracy (ZeroR = 0.613) is reported in the Results section to contextualize model performance.

- Input resolution: All images resized to 224 x 224 pixels.

- Normalization: ImageNet mean and standard deviation.

- Class mapping: Explicit rules for how MSLD v1.0 and v2.0 categories were mapped to the binary mpox/non-mpox labels.

Section 3.3 (Leakage control, grouping, and split policy) provides:

- Grouping: 617 filename-derived groups using a specified regular expression, with an average of 1.42 images per group and a maximum of 14.

- Near-duplicate audit: A 64-bit perceptual-hash (pHash) audit was conducted over all 876 original images. The audit identified 95 candidate near-duplicate pairs (Hamming distance <= 10); in every case, both members belonged to the same cross-validation fold, yielding zero cross-fold leaks.

- Augmentation control: Augmented images capped at 3 variants per group (citing Shorten and Khoshgoftaar 2019); original-image training ratio set to 0.70.

- Potential confounders: The Discussion acknowledges that source-level heterogeneity (different acquisition devices, lighting conditions, and racial diversity profiles between MSLD v1.0 and v2.0) is a primary driver of fold-level variability.

EDITOR CONCERN 4: Experimental Details Insufficient for Reproducibility

------------------------------------------------------------------------

"Critical missing information includes: preprocessing details, augmentation details, training details, fine-tuning details, hardware/software, hyperparameter selection."

RESPONSE:

All requested experimental details are now fully documented:

- Preprocessing: Images resized to 224 x 224, normalized with ImageNet mean [0.485, 0.456, 0.406] and standard deviation [0.229, 0.224, 0.225]. Augmented images excluded from fold construction and from all test/validation evaluations.

- Augmentation: Augmented variants used only during training, capped at 3 per group. Online augmentation parameters (MixUp alpha, CutMix alpha, RandAugment) are reported per backbone in Table 3.

- Training: Optimizer, learning rate, weight decay, label smoothing, loss function (cross-entropy or focal), scheduler, warmup epochs, gradient clipping, and drop-path rate are reported per backbone in Tables 2-3. These were selected by an NSGA-II multi-objective hyperparameter search (840 trials, 6 epochs each).

- Fine-tuning: After main training, a 5-epoch fine-tuning phase at lr = 1e-5 was applied on original-only training data to address the augmented-to-original domain shift. All layers were unfrozen. MixUp and CutMix were disabled during fine-tuning.

- Early stopping: Patience = 4 epochs, monitored metric = f1_at_precision on the validation set.

- Hardware: NVIDIA A100 (40 GB) GPU for training; NVIDIA GeForce RTX 4060 Laptop GPU for TTA ablation re-evaluation (Section 3.9).

- Software: Python 3.10, PyTorch 2.1, torchvision 0.16, Optuna 3.4, scikit-learn 1.3, SciPy 1.11, ImageHash.

- Seeds: 12345-12349 (one per fold), fixed for reproducibility.

- Compute budget: 15-25 GPU-hours total.

- Pretrained weights: torchvision ImageNet-1K V1 variants for all backbones (Section 3.9).

- Model complexity: Trainable parameters and GFLOPs (computed via the torchprofile library) are reported in Table 4.

EDITOR CONCERN 5: Near-Perfect Performance Raises Concerns

-----------------------------------------------------------

"EfficientNet achieving 99.64% validation accuracy with 100% precision and recall on a medical image classification task is extraordinarily high."

RESPONSE:

This concern has been fully resolved. The former manuscript's near-perfect results have been replaced with substantially lower and intentionally conservative results obtained under a stricter protocol:

- Weighted ensemble: F1 = 0.8334 +/- 0.0432, AUC = 0.9388 +/- 0.0203

- Best single model (ConvNeXt-Tiny): F1 = 0.8159 +/- 0.0727, AUC = 0.9284 +/- 0.0303

These results are reported under 5-fold group-stratified cross-validation with original-only test evaluation, meaning no augmented images appear in test or validation sets. The abstract explicitly characterizes these as "deflated but more trustworthy reference values."

Five robustness analyses substantiate the integrity of these results:

(i) A perceptual-hash near-duplicate audit confirmed zero cross-fold data leakage across all 876 images (Section 3.3).

(ii) Pre- and post-calibration Expected Calibration Error (ECE) is quantified for every model in every fold, revealing both the benefits and limits of temperature scaling (Section 4.6, Table 5).

(iii) Per-fold decision thresholds are reported with explicit verification of whether precision (>= 0.80) and recall (>= 0.85) targets were met on the test partition (Section 4.7, Table 7).

(iv) A TTA ablation using the same saved checkpoints and identical data splits demonstrates that the reported results are not critically dependent on the test-time augmentation protocol; the ensemble F1 gain from TTA is +0.009 (Section 4.8, Table 8).

(v) Four complementary statistical tests (McNemar, permutation, Wilcoxon signed-rank, DeLong) with Bonferroni correction are applied to all pairwise model comparisons (Section 4.5).

Error analysis is provided through fold-wise confusion matrix components (Table 8, showing TP, FP, TN, FN per fold) and fold-specific confusion matrix, ROC, and precision-recall plots (Figures 1-4).

EDITOR CONCERN 6: Smart Clinic Concept Underdeveloped

------------------------------------------------------

"The concept of an unmanned smart clinic is underdeveloped. It is not integrated with the core analysis."

RESPONSE:

The smart clinic concept has been entirely removed from the revised manuscript. The paper is now purely a benchmarking study focused on methodological rigour in mpox skin-image classification. This decision was made because the clinic concept was a conceptual proposal without empirical validation, as the Editor correctly noted.

EDITOR CONCERN 7: Literature Comparison Table Problematic

----------------------------------------------------------

"The table does not indicate dataset sizes, class distributions, or validation methodologies, making comparisons meaningless."

RESPONSE:

Table 1 has been completely redesigned as a standardized longtable with five columns: Study, Task/dataset, Split/validation, Best reported result, and Comparability limitation. It now covers 12 peer-reviewed studies spanning 2022-2025. Each entry documents the dataset size, class structure, validation strategy, and best reported result, alongside a specific explanation of why direct comparison with the present benchmark is limited (e.g., "binary evaluation performed on an augmented four-class pool," "headline accuracy is a validation figure obtained during hyperparameter search," "model ranking reverses across datasets"). The present study is included as the final row with a gray highlight. This design ensures that readers can assess comparability rather than simply rank headline numbers.

EDITOR CONCERN 8: Writing and Presentation

-------------------------------------------

"The manuscript has significant presentation problems: redundancy, organization, figures, table formatting, references."

RESPONSE:

The manuscript has been completely rewritten and restructured:

- Structure: Introduction, Related work, Materials and methods (with 9 subsections), Results (with 9 subsections including new calibration, threshold, and TTA analyses), Discussion, Conclusion. No redundancy between sections.

- Figures: All figures are in PNG format with self-explanatory captions that expand all acronyms and explain color/linestyle legends. Confusion matrices, ROC curves, and precision-recall curves are presented for four representative models using different folds to avoid overemphasizing any single split.

- Tables: 9 tables, all using booktabs formatting (toprule, midrule, bottomrule) with no vertical rules. Wide tables use resizebox to prevent overflow. Statistical notation (mean +/- SD) is consistent throughout.

- References: 38 references, all integrated into the narrative with specific attribution. PLOS ONE Vancouver style (plos2025.bst). All entries include DOIs.

- Formatting: Line numbers and double spacing enabled. 10pt font, letterpaper, 1-inch margins.

EDITOR REQUIRED REVISION E: External Validation

-------------------------------------------------

"Test the models on an independent dataset not used in development. If external validation is not possible, explicitly discuss this as a major limitation."

RESPONSE:

External validation on an independent clinical cohort was not performed in this revision. While other public mpox-related image collections exist, these differ substantially from the present benchmark in class definitions, labeling protocols, image quality, and acquisition conditions. Using them as an external test set without careful harmonization would introduce confounds that undermine the interpretability of the comparison — precisely the problem this benchmark is designed to address. Rather than report a potentially misleading external validation on incompatible data, we chose to strengthen the internal evidence base through a comprehensive suite of robustness analyses and to identify external validation on a prospectively collected, protocol-matched clinical cohort as the highest priority for future work. This limitation is explicitly acknowledged in the Discussion as the first limitation listed. However, the revised manuscript compensates for this gap with a comprehensive suite of internal robustness analyses that collectively provide stronger evidence of methodological soundness than is typical of single-dataset benchmarks:

(i) Content-level near-duplicate auditing via perceptual hashing confirmed zero cross-fold leaks across 876 images (Section 3.3).

(ii) Pre- and post-calibration ECE was quantified for every model in every fold, revealing both the benefits and limits of temperature scaling (Section 4.6).

(iii) Per-fold decision thresholds were reported with explicit verification of whether precision and recall targets were met on the test partition (Section 4.7).

(iv) A TTA ablation using the same saved checkpoints and identical data splits demonstrated that the reported results are not critically dependent on the test-time augmentation protocol (Section 4.8).

(v) Four complementary statistical tests with Bonferroni correction were applied to all pairwise model comparisons within each fold (Section 4.5).

While these analyses do not substitute for external validation, they ensure that the reported performance estimates are internally consistent, auditable, and not inflated by methodological artefacts. External validation on a prospectively collected clinical cohort is identified as the highest priority for future work in both the Limitations paragraph and the Conclusion.

=========================================================

RESPONSE TO REVIEWER 1

=========================================================

REVIEWER 1: "There is no novelty in the methodology. This study is totally relied on established architectures without any significant advancement. The paper is not suitable for a reputable journal like plos one."

RESPONSE:

We respectfully acknowledge this concern and agree that the former manuscript failed to articulate a clear contribution. The revised manuscript has been completely reframed. The contribution is now explicitly methodological: a transparent benchmark

Attachment Submitted filename: response_to_reviewers.docx

# Editor report: Decision Letter 1

**DOI:** 10.1371/journal.pone.0352161.r003

**Authors:** Morufu Olalekan Raimi

31 May 2026

PONE-D-25-66974R1

AI-Driven Diagnosis of Monkeypox using Deep Learning Models

PLOS One

Dear Dr. Sadek,

Thank you for submitting your manuscript to PLOS ONE. After careful consideration, we feel that it has merit but does not fully meet PLOS ONE’s publication criteria as it currently stands. Therefore, we invite you to submit a revised version of the manuscript that addresses the points raised during the review process.

Please submit your revised manuscript by Jul 15 2026 11:59PM. If you will need more time than this to complete your revisions, please reply to this message or contact the journal office at plosone@plos.org. When you're ready to submit your revision, log on to https://www.editorialmanager.com/pone/ and select the 'Submissions Needing Revision' folder to locate your manuscript file.

Please include the following items when submitting your revised manuscript:

- A letter that responds to each point raised by the academic editor and reviewer(s). You should upload this letter as a separate file labeled 'Response to Reviewers'.
- A marked-up copy of your manuscript that highlights changes made to the original version. You should upload this as a separate file labeled 'Revised Manuscript with Track Changes'.
- An unmarked version of your revised paper without tracked changes. You should upload this as a separate file labeled 'Manuscript'.

If you would like to make changes to your financial disclosure, please include your updated statement in your cover letter. Guidelines for resubmitting your figure files are available below the reviewer comments at the end of this letter.

If applicable, we recommend that you deposit your laboratory protocols in protocols.io to enhance the reproducibility of your results. Protocols.io assigns your protocol its own identifier (DOI) so that it can be cited independently in the future. For instructions see: https://journals.plos.org/plosone/s/submission-guidelines#loc-laboratory-protocols. Additionally, PLOS ONE offers an option for publishing peer-reviewed Lab Protocol articles, which describe protocols hosted on protocols.io. Read more information on sharing protocols at https://plos.org/protocols?utm_medium=editorial-email&utm_source=authorletters&utm_campaign=protocols.

As the corresponding author, your ORCID iD is verified in the submission system and will appear in the published article. PLOS supports the use of ORCID, and we encourage all coauthors to register for an ORCID iD and use it as well. Please encourage your coauthors to verify their ORCID iD within the submission system before final acceptance, as unverified ORCID iDs will not appear in the published article. *Only*  the individual author can complete the verification step; PLOS staff *cannot*  verify ORCID iDs on behalf of authors.

We look forward to receiving your revised manuscript.

Kind regards,

Morufu Olalekan Raimi, Ph.D

Academic Editor

PLOS One

**Journal Requirements:**

1. If the reviewer comments include a recommendation to cite specific previously published works, please review and evaluate these publications to determine whether they are relevant and should be cited. There is no requirement to cite these works unless the editor has indicated otherwise.

2. Please review your reference list to ensure that it is complete and correct. If you have cited papers that have been retracted, please include the rationale for doing so in the manuscript text, or remove these references and replace them with relevant current references. Any changes to the reference list should be mentioned in the rebuttal letter that accompanies your revised manuscript. If you need to cite a retracted article, indicate the article’s retracted status in the References list and also include a citation and full reference for the retraction notice.

**Additional Editor Comments:**

PLOS ONE Editorial Decision

Manuscript ID: PONE-D-25-66974_R1

Title: AI-Driven Diagnosis of Monkeypox using Deep Learning Models

Authors: Aboshosha et al.

Editor: Dr. Morufu Olalekan Raimi

Decision: Minor Revision

**Summary of Evaluation**

The authors have submitted a substantially revised manuscript that directly and comprehensively addresses the concerns raised in the previous round of review. The original submission suffered from a lack of methodological novelty, inadequate dataset documentation, insufficient experimental detail, near perfect but implausible performance metrics, and an underdeveloped “smart clinic” concept. The revised version has been fundamentally restructured. The paper is now explicitly framed as a methodological benchmark rather than an architectural contribution. It provides a leakage aware, group stratified five fold cross validation protocol on a unified dataset from MSLD v1.0 and v2.0. The smart clinic concept has been removed, near perfect results replaced by conservative but credible estimates (ensemble F1 = 0.8334, AUC = 0.9388), and all previously missing experimental details (preprocessing, augmentation, hyperparameter search, fine tuning, hardware/software, seeds) are now fully documented. The authors also provide a perceptual hash near duplicate audit, calibration analysis, threshold verification, test time augmentation ablation, and multiple statistical tests with Bonferroni correction. Given the rigor of the revisions, the manuscript now meets PLOS ONE’s standards for methodological transparency and reproducibility. However, a few remaining issues require attention before final acceptance.

**Required Minor Revisions**

1. Title inconsistency

The main manuscript (pages 17 and 63) uses “Mpox” and “Monkeypox” interchangeably in the title and abstract. Please standardize to “Mpox” throughout, consistent with current WHO terminology.

2. Table numbering discrepancies

In the point by point response, the authors refer to “Table 3” for augmentation parameters and “Table 4” for complexity, but in the manuscript, augmentation hyperparameters appear in Table 5 and complexity in Table 7. Please reconcile all cross references in the response letter to match the final manuscript’s table numbering.

3. Figure 1 caption clarity

Figure 1 (dot and whisker plot) is well constructed, but the caption should explicitly state that points are mean values across five folds and whiskers represent ±1 standard deviation. This is implied but not fully spelled out.

4. External validation statement

The Discussion correctly lists the lack of external validation as a limitation. However, the Conclusion currently states that “future work should extend this line of research through externally validated datasets.” Please add a one sentence acknowledgment in the Abstract as well, e.g., “External clinical validation remains necessary before deployment.”

5. Minor typographical corrections

o Page 41, line 359: “originally test evaluation” → “original only test evaluation”

o Page 73, Table 3: “16 fold test time augmentation” row is correct, but the caption of Table 12 (page 82) mislabels TTA as “×16” – this is fine, but ensure consistent wording (“16 fold” vs. “×16”) across the manuscript.

**Editorial Comments**

The authors have done an exemplary job responding to the previous criticisms. The revised benchmark is transparent, reproducible, and appropriately conservative. The removal of the smart clinic concept was necessary and correct. The new results are credible and well supported by robustness analyses. The paper now makes a clear methodological contribution to a literature that has suffered from inconsistent evaluation practices. Once the above minor corrections are made, the manuscript will be acceptable for publication in PLOS ONE.

Final decision: Minor revision. No further peer review is anticipated, but the revised manuscript will be checked for compliance with the above items.

Dr. Morufu Olalekan Raimi

PLOS ONE Academic Editor

[Note: HTML markup is below. Please do not edit.]

[NOTE: If reviewer comments were submitted as an attachment file, they will be attached to this email and accessible via the submission site. Please log into your account, locate the manuscript record, and check for the action link "View Attachments". If this link does not appear, there are no attachment files.]

To ensure your figures meet our technical requirements, please review our figure guidelines: https://journals.plos.org/plosone/s/figures

You may also use PLOS’s free figure tool, NAAS, to help you prepare publication quality figures: https://journals.plos.org/plosone/s/figures#loc-tools-for-figure-preparation.

NAAS will assess whether your figures meet our technical requirements by comparing each figure against our figure specifications.

# Author comment: Author response to Decision Letter 2

**DOI:** 10.1371/journal.pone.0352161.r004

3 Jun 2026

RESPONSE TO THE ACADEMIC EDITOR

Manuscript ID: PONE-D-25-66974_R1

Title: AI-Driven Diagnosis of Mpox using Deep Learning Models

Authors: Aboshosha et al.

Academic Editor: Dr. Morufu Olalekan Raimi

Decision: Minor Revision

------------------------------------------------------------

Dear Dr. Raimi,

We thank you for the careful and constructive evaluation of our revised manuscript and for the positive assessment of the methodological restructuring. We are grateful for the recommendation toward acceptance. We have addressed each of the required minor revisions point by point below. For every item we indicate the action taken and the location in the revised manuscript. The editor's comments are reproduced first, followed by our response.

Throughout this letter, all table and figure references use the final manuscript numbering, which we have re-verified against the compiled document. For convenience, the final table numbering is summarized at the end of this letter.

------------------------------------------------------------

RESPONSE TO JOURNAL REQUIREMENTS

Reference list completeness and retraction check. We have reviewed the full reference list for completeness and accuracy. Every in-text citation resolves to a complete bibliographic entry (author, title, venue, year, and DOI where applicable), and there are no orphaned, duplicated, or missing references. We additionally checked the cited DOIs against Crossref and the Retraction Watch database; to the best of our knowledge none of the cited works has been retracted or carries an expression of concern. Consequently, no references were removed or replaced on these grounds, and no retraction notices are required. Should any item be identified as retracted during production, we will indicate its retracted status in the reference list and add a full citation to the corresponding retraction notice, as required.

------------------------------------------------------------

REQUIRED MINOR REVISIONS

------------------------------------------------------------

COMMENT 1 - Title inconsistency

Editor: The main manuscript uses "Mpox" and "Monkeypox" interchangeably in the title and abstract. Please standardize to "Mpox" throughout, consistent with current WHO terminology.

Response: We have standardized the disease terminology to "Mpox" in line with current WHO nomenclature.

- The title now reads "AI-Driven Diagnosis of Mpox using Deep Learning Models."

- The abstract and body text now use "mpox" consistently, including the previously inconsistent descriptive passages in the Related Work section (e.g., "mpox detection from skin lesion images" and "mpox recognition from visual images") and the class listing in the comparison table.

For accuracy, we have retained "monkeypox" only where it is scientifically correct or verbatim, namely: (i) the formal virus name "monkeypox virus (MPXV)," which WHO continues to use for the pathogen; (ii) literal dataset folder labels reproduced from the public MSLD repositories (e.g., "Monkey Pox", "Monkeypox"), which must remain verbatim for reproducibility; (iii) the proper model name "MonkeyNet"; and (iv) bibliographic citation keys. We trust this distinction is consistent with the editor's intent.

------------------------------------------------------------

COMMENT 2 - Table numbering discrepancies

Editor: In the point-by-point response, the authors refer to "Table 3" for augmentation parameters and "Table 4" for complexity, but in the manuscript, augmentation hyperparameters appear in Table 5 and complexity in Table 7. Please reconcile all cross-references in the response letter to match the final manuscript's table numbering.

Response: We apologize for the inconsistency in the previous response letter. We have re-verified the table numbering in the final compiled manuscript and corrected all cross-references accordingly. In the final manuscript:

- Augmentation and regularization hyperparameters appear in Table 5 ("Selected backbone-specific augmentation and regularization hyperparameters").

- Model complexity appears in Table 7 ("Model complexity of the evaluated backbones").

All table references in this response letter now match the final manuscript numbering (see the summary at the end of this letter). No further table-numbering inconsistencies remain.

------------------------------------------------------------

COMMENT 3 - Figure 1 caption clarity

Editor: Figure 1 (dot and whisker plot) is well constructed, but the caption should explicitly state that points are mean values across five folds and whiskers represent +/- 1 standard deviation.

Response: We have revised the caption of Figure 1 to state this explicitly. It now reads:

"Cross-validation performance summary of the evaluated methods across five folds. Each point represents the mean value across the five cross-validation folds, and the whiskers represent +/- 1 standard deviation around that mean. Methods are ordered by mean F1-score."

------------------------------------------------------------

COMMENT 4 - External validation statement

Editor: The Discussion correctly lists the lack of external validation as a limitation. However, the Conclusion currently states that "future work should extend this line of research through externally validated datasets." Please add a one-sentence acknowledgment in the Abstract as well, e.g., "External clinical validation remains necessary before deployment."

Response: We have added the requested acknowledgment to the end of the Abstract, phrased as a logically linked closing statement rather than a standalone sentence so that it follows naturally from the methodological framing. The Abstract now closes with:

"...while highlighting the limitations of current public skin-image data. Accordingly, these results should be interpreted as a reproducible reference benchmark rather than a clinically validated diagnostic tool, and external clinical validation remains necessary before deployment."

This complements the existing statements in the Discussion and Conclusion.

------------------------------------------------------------

COMMENT 5 - Minor typographical corrections

Editor (a): "originally test evaluation" -> "original only test evaluation".

Response: The phrasing throughout the manuscript now reads "original-only test evaluation" (e.g., in the Abstract, Related Work, Methods, Results, and Discussion). The flagged typographic form is no longer present.

Editor (b): Ensure consistent wording ("16 fold" vs. "x16") for test-time augmentation across the manuscript.

Response: We have standardized the wording to "16-fold" throughout. Specifically:

- The shared training-and-evaluation settings table now lists the test-time augmentation value as "16-fold."

- The TTA ablation table caption now reads "Effect of 16-fold test-time augmentation (TTA) on mean F1-score and AUC across five folds," and its column header reads "TTA on (16-fold)."

- The running text already used "16-fold," so the terminology is now uniform.

------------------------------------------------------------

SUMMARY OF FINAL MANUSCRIPT TABLE NUMBERING (for reference)

Table 1 - Standardized comparison of representative peer-reviewed mpox skin-image studies

Table 2 - Unified dataset construction rules

Table 3 - Shared training and evaluation settings (includes the TTA = 16-fold row)

Table 4 - Selected backbone-specific optimization and loss hyperparameters

Table 5 - Selected backbone-specific AUGMENTATION and regularization hyperparameters

Table 6 - Fold-wise test performance (mean +/- SD)

Table 7 - Model COMPLEXITY of the evaluated backbones

Table 8 - Mean confusion-matrix components

Table 9 - Normalized ensemble weights per fold

Table 10 - Expected Calibration Error before/after temperature scaling

Table 11 - Validation-selected decision thresholds and test precision/recall

Table 12 - Effect of 16-fold test-time augmentation (TTA) on F1 and AUC

Figure 1 is the dot-and-whisker cross-validation performance summary.

------------------------------------------------------------

We believe these revisions fully address the remaining points raised. We thank you again for the constructive review, which has further improved the clarity and consistency of the manuscript.

Sincerely,

Ibrahim Sadek, on behalf of all co-authors

Corresponding author

Attachment Submitted filename: Response_to_Editor_R2.docx

# Editor report: Decision Letter 2

**DOI:** 10.1371/journal.pone.0352161.r005

**Authors:** Morufu Olalekan Raimi

7 Jun 2026

AI-Driven Diagnosis of Mpox using Deep Learning Models

PONE-D-25-66974R2

Dear Author,

We’re pleased to inform you that your manuscript has been judged scientifically suitable for publication and will be formally accepted for publication once it meets all outstanding technical requirements.

Within one week, you’ll receive an e-mail detailing the required amendments. When these have been addressed, you’ll receive a formal acceptance letter and your manuscript will be scheduled for publication.

An invoice will be generated when your article is formally accepted. Please note, if your institution has a publishing partnership with PLOS and your article meets the relevant criteria, all or part of your publication costs will be covered. Please make sure your user information is up-to-date by logging into Editorial Manager at Editorial Manager® and clicking the ‘Update My Information' link at the top of the page. For questions related to billing, please contact billing support.

If your institution or institutions have a press office, please notify them about your upcoming paper to help maximize its impact. If they’ll be preparing press materials, please inform our press team as soon as possible -- no later than 48 hours after receiving the formal acceptance. Your manuscript will remain under strict press embargo until 2 pm Eastern Time on the date of publication. For more information, please contact onepress@plos.org.

Kind regards,

Morufu Olalekan Raimi, Ph.D

Academic Editor

PLOS One

Additional Editor Comments (optional):

PLOS ONE Editorial Decision

Manuscript ID: PONE-D-25-66974_R2

Title: AI-Driven Diagnosis of Mpox using Deep Learning Models

Authors: Aboshosha et al.

Editor: Dr. Morufu Olalekan Raimi

Decision: Accept

Editorial Assessment

The authors have submitted a revised manuscript (R2) that fully addresses all five required minor revisions from the previous decision letter (R1). I have verified each point:

1. Title terminology – Standardized to “Mpox” throughout, with appropriate retention of “monkeypox virus (MPXV)” and dataset folder names. Compliant.

2. Table numbering – The response letter now correctly references Table 5 (augmentation) and Table 7 (complexity). No remaining discrepancies.

3. Figure 1 caption – Explicitly states that points are mean values across five folds and whiskers represent ±1 standard deviation. Sufficiently clear.

4. External validation statement – Added to the Abstract as requested, closing with: “Accordingly, these results should be interpreted as a reproducible reference benchmark rather than a clinically validated diagnostic tool, and external clinical validation remains necessary before deployment.” Acceptable.

5. Typographical corrections – “original-only test evaluation” now appears consistently; test-time augmentation terminology standardized to “16-fold” across all tables, captions, and running text.

No new issues have been introduced. The manuscript remains methodologically rigorous, transparent, and appropriately conservative in its claims. The benchmark serves as a valuable reference for the mpox imaging community.

Final Decision: Accept. No further revisions required.

Dr. Morufu Olalekan Raimi

Academic Editor, PLOS ONE

Reviewers' comments:

# Editor report: Acceptance letter

**DOI:** 10.1371/journal.pone.0352161.r006

**Authors:** Morufu Olalekan Raimi

PONE-D-25-66974R2

PLOS One

Dear Dr. Sadek,

I'm pleased to inform you that your manuscript has been deemed suitable for publication in PLOS One. Congratulations! Your manuscript is now being handed over to our production team.

At this stage, our production department will prepare your paper for publication. This includes ensuring the following:

* All references, tables, and figures are properly cited

* All relevant supporting information is included in the manuscript submission,

* There are no issues that prevent the paper from being properly typeset

You will receive further instructions from the production team, including instructions on how to review your proof when it is ready. Please keep in mind that we are working through a large volume of accepted articles, so please give us a few days to review your paper and let you know the next and final steps.

Lastly, if your institution or institutions have a press office, please let them know about your upcoming paper now to help maximize its impact. If they'll be preparing press materials, please inform our press team within the next 48 hours. Your manuscript will remain under strict press embargo until 2 pm Eastern Time on the date of publication. For more information, please contact onepress@plos.org.

You will receive an invoice from PLOS for your publication fee after your manuscript has reached the completed accept phase. If you receive an email requesting payment before acceptance or for any other service, this may be a phishing scheme. Learn how to identify phishing emails and protect your accounts at https://explore.plos.org/phishing.

If we can help with anything else, please email us at customercare@plos.org.

Thank you for submitting your work to PLOS ONE and supporting open access.

Kind regards,

PLOS ONE Editorial Office Staff

on behalf of

Prof Morufu Olalekan Raimi

Academic Editor

PLOS One
